# Electrical Signals, Plant Tolerance to Actions of Stressors, and Programmed Cell Death: Is Interaction Possible?

**DOI:** 10.3390/plants10081704

**Published:** 2021-08-19

**Authors:** Ekaterina Sukhova, Vladimir Sukhov

**Affiliations:** Department of Biophysics, N.I. Lobachevsky State University of Nizhny Novgorod, 603950 Nizhny Novgorod, Russia; n.catherine@inbox.ru

**Keywords:** electrical signals, physiological responses, programmed cell death, tolerance, stressors, plants

## Abstract

In environmental conditions, plants are affected by abiotic and biotic stressors which can be heterogenous. This means that the systemic plant adaptive responses on their actions require long-distance stress signals including electrical signals (ESs). ESs are based on transient changes in the activities of ion channels and H^+^-ATP-ase in the plasma membrane. They influence numerous physiological processes, including gene expression, phytohormone synthesis, photosynthesis, respiration, phloem mass flow, ATP content, and many others. It is considered that these changes increase plant tolerance to the action of stressors; the effect can be related to stimulation of damages of specific molecular structures. In this review, we hypothesize that programmed cell death (PCD) in plant cells can be interconnected with ESs. There are the following points supporting this hypothesis. (i) Propagation of ESs can be related to ROS waves; these waves are a probable mechanism of PCD initiation. (ii) ESs induce the inactivation of photosynthetic dark reactions and activation of respiration. Both responses can also produce ROS and, probably, induce PCD. (iii) ESs stimulate the synthesis of stress phytohormones (e.g., jasmonic acid, salicylic acid, and ethylene) which are known to contribute to the induction of PCD. (iv) Generation of ESs accompanies K^+^ efflux from the cytoplasm that is also a mechanism of induction of PCD. Our review argues for the possibility of PCD induction by electrical signals and shows some directions of future investigations in the field.

## 1. Introduction

Plants are affected by numerous environmental factors including abiotic and biotic stressors. The actions of many stressors (e.g., low or high temperatures, mechanical damages, drought, insect attacks, damage by pathogens, excess light, and many others) can be spatially heterogenous. This means that long-distance stress signals, which induce systemic adaptation responses and coordinate physiological changes in different parts of plants, are necessary [1,2,3,4,5,6,7,8,9,10]. There are different types of long-distance stress signals [1,2,3,4,5,6,7,8,9,10] including numerous chemical signals (phytohormones [1,11,12,13], small proteins and peptides [7,14], glutamate [9,15], and others), reactive oxygen species (ROS) waves [16,17,18,19], Ca^2+^ waves [20,21,22,23], hydraulic waves [3,24,25,26,27], and electrical signals (ESs) [8,10,28,29,30,31,32,33,34,35,36,37]. 

ESs can propagate in seconds or minutes after the local actions of stressors because their velocities are typically from about several hundred µm s^−1^ to several cm s^−1^ and more [8,29,34,38]. This means that electrical signals (along with hydraulic signals) participate in the induction of early systemic physiological responses after the local actions of stressors [8,34]. It is also considered that ESs can interact with other types of long-distance stress signals including hormonal signals [10,11], ROS waves [17,19], hydraulic waves [24,25], and Ca^2+^ waves [20,32]. Additionally, there are numerous works (e.g., see reviews [8,29,30,31,32,33,34,35,36]) which show the rapid influence of ESs on different physiological processes. It is considered that the result of ES-induced physiological changes is increased plant tolerance to the actions of stressors [8,34,36,39,40] that is supported by the positive influence of electrical signals on the tolerance shown in experimental works [41,42,43,44,45,46,47]. The relation between ESs and tolerance to stressors can be the basis of the estimation of the action of stressors on plants through measurements of electrical activity [47,48,49,50,51,52,53,54,55,56,57]. 

However, increasing the total plant tolerance to stressors is a complex process which can include damage to specific processes, cells, or parts of plants. Programmed cell death (PCD) is a well-known process which regulates the development of a living organism or protects it under the actions of stressors by inducing the death of certain cells [58,59]. In particular, in plants, stressors with high intensities are known to induce PCD through the production of ROS [59,60,61,62,63,64], synthesis of some phytohormones [62,65,66,67], K^+^ efflux from the cytoplasm [68,69,70,71,72,73], and other mechanisms. The noted processes seem to be similar to physiological changes (suppression of photosynthetic dark reactions, which is likely to cause ROS production; increase in respiration; production of jasmonic acid (JA) and other stress phytohormones; K^+^ leakage) which accompany ESs [8,34]. Considering the similarity, we hypothesize that ESs can be interconnected with PCD in plant cells. Our review is devoted to the analysis of this hypothesis based on knowledge about electrical signaling in plants and PCD. 

## 2. Electrical Signals in Plants: Types and Mechanisms

Unlike animals, where only action potential (AP) is observed, several types of long-distance electrical signals can be observed in plants [8,34,35,36]. In higher plants, there are AP [28,29], variation potential (VP) [31,34,74], and system potential (SP) [75,76]. Stressors can also induce subthreshold local electrical responses in plants [8,36]; however, their passive electrotonic propagation is strongly limited (several mm), and we will not analyze these responses further in this review. 

### 2.1. Action Potential

An AP is a short-term depolarization spike including two phases (depolarization and repolarization) [28,35,77,78] which is induced by non-damaging irritations (e.g., electrical current [79,80,81,82,83], cooling [41,79,84], touch [85,86,87], initiation or termination of illumination [88,89]). The generation of an AP is in accordance with the “all-or-none law” [8,28,35]; it is a self-propagating signal with typical velocities ranging from 1 mm s^−1^ to 10 cm s^−1^ in different plants [8,38,77,80]. A typical AP in plants (excluding action potentials in carnivorous plants) has a long-term refractory period equaling 0.5–20 min (absolute refractory period) and 2–300 min (relative refractory period) [8,80].

AP generation is initiated by activation of potential-dependent Ca^2+^ channels at the overthreshold depolarization of the electrical potential in the plasma membrane (the channels are not yet associated with any specific gene product [73]) and an increasing concentration of calcium ions in the cytoplasm [8,28,29,35,90,91]. An increase in the Ca^2+^ concentration activates Ca^2+^-dependent anion channels [28,90] (probably QUAC1 and/or SLAC1 channels [33,91]) and inactivates H^+^-ATP-ase [35,92,93] in the plasma membrane. Large depolarization inactivates Ca^2+^ channels, thereby decreasing the cytoplasmic concentration of calcium ions, and activates outward-rectifying K+ channels [90] (probably GORK [33,91]); the latter channels participate in the repolarization. A decrease in the Ca^2+^ concentration inactivates anion channels and activates H^+^-ATP-ase in the plasma membrane [35,91]; both processes also contribute to AP repolarization. 

AP propagation is an active process which is mediated by plant vascular bundles [8,28]. There are two potential ways of AP propagation: (i) propagation through the symplast of parenchyma cells in these bundles [8,35], and (ii) propagation through sieve elements [29,94,95]. It is also possible that both ways can simultaneously participate in the propagation of AP [8].

### 2.2. Variation Potential

VP is a long-term depolarization signal (minutes, tens of minutes, and even hours) which is considered to be a unique ES in higher plants [8,31,74]. A variation potential is induced by local damages (e.g., burning [11,25,96,97,98], gradual heating [47,99,100], or wounding [11,101]); it has some specific properties [8,31]. (i) VP has an irregular shape. It includes a basic long-term depolarization and, possibly, an additional fast depolarization and/or “AP-like” spikes [8,31,34,35,74,102]. (ii) The amplitude, shape, and velocity of the propagation of VP can be dependent on the distance from a damaged zone or the intensity of stimulus [8,31,74,99,100,103,104]. The velocity of the propagation of VP ranges from 200 µm s^−1^ to about 2 cm s^−1^ [8]. (iii) VP can propagate at the refractory period of AP; it is able to pass through inactive and dead tissues [8,29]. Considering its properties, VP is probably a local electrical response induced by the propagation of a non-electrical long-distance signal [8,29,31,34,35].

The generation of VP includes two groups of mechanisms [8,31]. The initial fast depolarization and AP-like spikes are very probably local action potentials induced by the long-term depolarization [31,35,105]. The mechanisms of these responses are similar to AP mechanisms (see Section 2.1) [31]: the depolarization transiently activates potential-dependent Ca^2+^ channels and induces an increase in the Ca^2+^ concentration, where this increased concentration transiently activates Ca^2+^-dependent anion channels and inactivates H^+^-ATP-ase; K^+^ efflux through outward-rectifying K^+^ channels participates in the repolarization of the AP-like spikes. The duration of the initial fast depolarization and quantity of AP-like spikes depend on the amplitude and duration of the long-term depolarization [31,105]. This means that these components of VP can induce large changes in the cytoplasmic and apoplastic concentrations of Cl^-^ and K^+^ because the generation of AP is accompanied by changes in these concentrations [90,93]; the magnitudes of the Cl^-^ and K^+^ concentration changes should be indirectly dependent on the parameters of the long-term depolarization.

In contrast, long-term depolarization is a unique electrical response induced by non-electrical signals [8,31]. The generation of long-term depolarization is related to a transient inactivation of H^+^-ATP-ase in the plasma membrane [8,29,31,74,106]. This inactivation is induced by a Ca^2+^ influx through mechanosensitive and/or ligand-dependent calcium channels [8,31,34,35,107] (possibly GLR, MSL, and CNGC [19]), which increase the concentration of calcium ions in the cytoplasm. It is considered [31] that changes in the Cl^−^ and K^+^ concentrations are rather weak at the generation of long-term depolarization only (if the initial fast depolarization and/or AP-like spikes are absent).

There are chemical and hydraulic hypotheses about the mechanisms of the propagation of VP [8,31,34,35]. In accordance with the chemical hypothesis, long-term depolarization is a local electrical response induced by a specific chemical agent (wound substance) which propagates from the damaged zone and activates ligand-dependent calcium channels [8,31]. The wound substance remains unclear; however, H_2_O_2_ seems to be a likely candidate [31]. There are modified variants of the chemical hypothesis including “hydraulic dispersion” (propagation of the wound substance in a water mass flow induced by increased pressure in the damaged zone [24,108,109]) and “turbulent diffusion” (propagation of the wound substance in xylem on the basis of diffusion accelerated by a turbulent water flow [35,104,105,110]).

The hydraulic hypothesis is an alternative hypothesis of VP propagation [8,31]. The hypothesis supposes that local damages induce the propagation of waves of an increased pressure through the plant body, activation of mechanosensitive calcium channels, and an increase in the Ca^2+^ concentration in the cytoplasm [25,31,74,103,111].

It should be noted that both the chemical and hydraulic hypotheses suppose a key role of Ca^2+^ influx in the induction of changes in the membrane potential (mainly through inactivation of H^+^-ATP-ase) [8,31]. This means that the mechanisms of VP generation can be the same at different mechanisms of VP propagation. Moreover, it cannot be fully excluded that VP propagation is simultaneously based on different mechanisms (e.g., hydraulic and ROS waves). 

### 2.3. System Potential

SP are long-term hyperpolarization signal in higher plants [75,76], which was shown in 2009. System potential is still weakly investigated. Transient activation of H^+^-ATP-ase in the plasma membrane is considered to be the main mechanism of SP generation [75]; however, there are results [112] supporting the participation of Ca^2+^ and K^+^ channels in this generation. An SP is considered to be a self-propagating signal [75]. It is known that an SP is accompanied by a decrease in the Ca^2+^ concentration in the apoplast [75]. On the basis of these facts, we earlier hypothesized [8] that SP propagation can be related to the propagation of waves of the decreased Ca^2+^ concentration.

### 2.4. Electrical Signals and ROS Waves

ROS waves are waves of an increased ROS concentration which can be induced by the actions of stressors and propagate through the plant body [4,17,19,39,40]. In accordance with the hypothesis by Mittler and co-workers [17,19,32,40], the propagation of ROS waves is based on the secondary production of ROS and an increase in the Ca^2+^ concentration in the cytoplasm: H_2_O_2_ is transported into the cytoplasm through aquaporins in the plasma membrane (plasma membrane-intrinsic protein channels, PIP2) and activates Ca^2+^ channels in the plasma membrane and/or tonoplast; the increase in the Ca^2+^ concentration activates the respiratory burst oxidase homolog D (RBOHD) in the plasma membrane and, thereby, stimulates the production of a superoxide radical; finally, the superoxide radical is transformed into H_2_O_2_, which can be transported into cells again. There are two potential ways of propagation of ROS waves [19,40]: apoplastic transmission of H_2_O_2_ from cell to cell, and symplastic transmission of the systemic signal through plasmodesmata, which can also induce an increase in the Ca^2+^ concentration and ROS production in neighboring cells.

The propagation of ROS waves is considered to be related to the propagation of ESs [32,40]. This hypothesis is in good accordance with the key role of the increase in the Ca^2+^ concentration in the generation of AP and VP [8,29,30,31,35]; it can be supposed that the increase in the Ca^2+^ concentration, which accompanies the ROS wave propagation, can induce the generation of electrical responses in the cells. The mechanism of AP propagation has been well investigated (see Section 2.1, works [8,28,29,35,94,95]); participation of ROS waves in this propagation seems unlikely. SP is caused by transient activation of H^+^-ATP-ase in the plasma membrane [75,76]; this means that an ROS wave-related increase in the concentration of Ca^2+^, which should inactivate this transporter [31,35,92,93], cannot be a mechanism of SP, too.

In contrast, interaction between VP propagation and ROS waves seems to be probable. In particular, long-term ESs with an irregular shape can be observed at the propagation of ROS waves [39]. The durations of increases in the ROS concentration at ROS waves can be equal to tens of minutes [39], similar to the durations of VPs [8]. The velocities of ROS waves induced by different stimuli can be 0.4–1.4 mm s^−1^ [40]; the velocities of VP propagation can be similar (less than 1 mm s^−1^ under the local crush, and about 2 mm s^−1^ under the local heating [99]). Moreover, some works [99] showed that the velocity of VP propagation can be approximately constant regardless of the stimulus (e.g., after the crush and heating in pea seedlings). This result is in good accordance with the self-propagating mechanism of the propagation of ROS waves [40].

Results have shown that ROS waves can be considered as one of the potential mechanisms of VP. However, VP properties, which are not in accordance with the properties of ROS waves, can also be observed: these are the high velocities of VP propagation (e.g., up to 20 mm s^−1^ [8]) and the decrease in the amplitude and/or the VP velocity with the increase in the distance from the damaged zone [31,47,99]. Our earlier theoretical analysis [99] showed that the results can be explained by the complex mechanism of VP propagation (e.g., combination of ROS waves with the turbulent diffusion of H_2_O_2_). Induction of VP on the basis of the combination of ROS waves and hydraulic signals [8,31,34,35,103,111] is also possible. 

## 3. Physiological Roles of Electrical Signals

### 3.1. Brief Phenomenology of Influence of Electrical Signals on Physiological Processes

It is well known that ESs participate in the induction of movement in some plants [29,30,77,113] including the movements of leaves in Mimosa, traps in carnivorous plants, filaments of stamens, and stigmas of pistils of Berberis. The mechanisms of ES-regulated movements are actively investigated (e.g., the trap control by ESs in *Dionaea muscipula* [86,87,114,115]). However, ESs can induce numerous physiological responses in plants without moving organs [8].

#### 3.1.1. Gene Expression

The expression of defense genes is an important target of ESs in plants [8]. It is well known that ESs can stimulate the expression of the genes of the proteinase inhibitor 1 and 2 (pin1 and pin2) [11,116,117,118,119] and anti-insect vegetative storage protein 2 (vsp2) [101], which protect plants against insect attacks. The expression of other genes (e.g., those encoding calmodulins [120,121] or the chloroplast mRNA-binding protein [122]) can also be stimulated by ESs. It is important that these changes in the expression of genes can be quickly induced after the propagation of ESs (e.g., the increase in the expression of pin2 genes is initiated within 15 min after stimulation [119,121]).

#### 3.1.2. Phytohormone Production 

Stimulation of the production of some stress phytohormones is another response induced by ESs [8,10]. It is known [11,86,87,96,97,100,101,123,124] that ESs can increase the production of abscisic, jasmonic, and salicylic acids (ABA, JA, and SA, respectively) in plant leaves. The increase can be induced for 10–20 min after stimulation [96,97,100,124]; the duration of the response can be from about 1–2 [100,124] to, at least, 6 h [11,125,126]. An increase in ethylene production in plant leaves is another result of ES propagation [127]. 

#### 3.1.3. Photosynthesis

The effect of ESs on photosynthetic processes is complex [8,34]. First, it has been shown that ESs decrease mesophyll conductivity for CO_2_ [128] that suppresses photosynthetic dark reactions and, thereby, limits a linear electron flow and stimulates a cyclic electron flow around photosystem I (PSI) in the electron transport chain of chloroplasts [96,97,98,99,129,130,131,132]. The increase in the non-photochemical quenching of chlorophyll fluorescence (NPQ) is another result of the CO_2_ assimilation drop [129,130,131,132]; in particular, stimulation of the energy-dependent component of NPQ was shown in our earlier work [133]. Second, ESs can further decrease the linear electron flow and increase the cyclic electron flow and NPQ under low CO_2_ concentrations and suppression of photosynthetic dark reactions [43,131,132,134]. This means [34] that ESs can additionally influence photosynthetic electron flows without changes in photosynthetic dark reactions. Third, ESs can induce weak changes in the distribution of light energy between photosystems and stimulate light absorption by photosystem II (PSII) [104]. It is important that the photosynthetic changes include two components [8,35]: a fast photosynthetic inactivation, which is initiated within about 1 min after the propagation of ESs and is observed for about 5–10 min, and a long-term photosynthetic inactivation, which is formed for 15–30 min and can be observed for hours. It should also be noted that ES-induced photosynthetic changes (mainly inactivation of photosynthetic dark reactions and a decrease in ATP consumption) increase the ATP content in leaves [135]. Results have shown that photosynthesis is an important target of electrical signals in plants [8,34].

#### 3.1.4. Respiration

It is known that ESs stimulate respiration in plants [130,131,135,136,137,138]. It is probable that the activation is related to ES-induced stimulation of alternative respiratory ways (e.g., stimulation of rotenone-insensitive alternative NADPH dehydrogenases) [138]. However, the ES-induced activation of respiration is strongly related to a transient increase in the ATP content in leaves [135]; this result rather supports the influence of electrical signals on the basic respiratory way.

#### 3.1.5. Phloem Mass Flow

A decrease in phloem mass flow is also induced by electrical signals [8,95]. The decrease is probably based on several processes. First, ESs induce unloading of phloem in plant leaves [139,140]. Second, ESs induce fast suppression of the phloem mass flow (from 15–45 s to 7–15 min after induction of ESs) [141,142,143]. Third, ESs can induce callose deposition which causes sieve plate occlusion and suppresses the phloem mass flow; the occlusion is formed for 15–25 min and remains for 1–3 h after induction of ESs [141,142,143]. It is probable that the intensity of irritation and the type of ES (AP or VP) influence the duration of suppression of the phloem mass flow [95,144].

#### 3.1.6. Transpiration

There are numerous works [11,44,96,97,131,145,146,147,148,149,150,151,152] which show that local irritations of plants induce changes in transpiration. The changes can be complex including a first fast decrease in transpiration (about 5 min after local damage) and its subsequent increase (about 15 min) and second long-term decrease (30–50 min) [152]; the magnitudes of the components of changes in transpiration are dependent on the humidity of the air. The fast decrease and increase in transpiration are weakly related to parameters of ESs [152]; in contrast, the long-term decrease is strongly related to these parameters. This means that fast changes in transpiration are probably directly caused by hydraulic waves; the long-term changes are caused by electrical signals.

#### 3.1.7. Plant Growth

Plant growth can be also affected by ESs [8,29,30]. It is known that ESs can decrease the growth processes; the duration of the effect can range from about 5 min (AP) [153] to tens of minutes (VP) [106].

#### 3.1.8. Leaf Reflectance 

We earlier showed that ESs influence leaf reflectance in plants [133,154,155,156], changing different reflectance indices (e.g., photochemical reflectance index or water index). The reflectance changes are results of specific ES-induced physiological responses (the photochemical reflectance index is related to photosynthetic processes [157,158,159,160,161,162], while the water index is related to the water content [156,163]); i.e., they can potentially be used for remote and proximal sensing of physiological responses induced by ESs.

### 3.2. Potential Mechanisms of Induction of Physiological Responses by Electrical Signals 

It is considered that the mechanisms of ES-induced physiological changes are mainly based on changes in ion concentrations accompanying the generation of electrical signals [8]; however, other mechanisms are also possible.

#### 3.2.1. Changes in Plasma Membrane H^+^-ATP-ase Activity and Intra- and Extracellular pH 

Changes in the activity of H^+^-ATP-ase in the plasma membrane likely represent the general mechanism of generation of ESs (its inactivation during AP [92,93] and VP [29,31,74,106] generation and its activation during SP generation [75,76]). In particular, the H^+^-ATP-ase inactivation accompanying AP [92,93] and VP [29,31,74,106] generations induced alkalization of the apoplast and acidification of the cytoplasm [90,92,134,150,164] which can influence physiological processes.

The participation of the inactivation of H^+^-ATP-ase and pH changes in the fast photosynthetic inactivation induced by ESs has been well investigated [8,35]. There are several points supporting this mechanism. (i) Modification of the activity of H^+^-ATP-ase strongly influences the magnitude of the fast photosynthetic inactivation [165,166]. (ii) The ES-induced decrease in photosynthetic CO_2_ assimilation is strongly correlated with the pH increase in the apoplast [167,168]. Additionally, our experimental [169] and theoretical [170] investigations showed that an increase in the apoplastic pH should decrease CO_2_ flux into cells and suppress photosynthetic dark reactions. (iii) The ES-induced increase in NPQ is strongly correlated with the pH increase in the cytoplasm [167,168]; generation of ESs causes acidification of the stroma and lumen of chloroplasts [171]. (iv) Artificial inactivation of H^+^-ATP-ase [166] or induction of a proton influx [134] causes photosynthetic changes which are similar to the fast changes induced by ESs. (v) The artificial decrease in the pH in medium for chloroplasts [134,150,172] or in perfused cells [173] induces photosynthetic changes which are similar to the fast changes induced by ESs. Results showed [8,35] that there are at least two mechanisms of these ESs’ effects on photosynthetic processes: the increase in the apoplastic pH, which suppresses CO_2_ flux into cells (probably through changes in the CO_2_/HCO_3_^-^ ratio [172] and modifications of activity of aquaporins transmitting CO_2_ [128]), and the decrease in the cytoplasmic pH and thereby the pH in the stroma and lumen, which directly affect photosynthetic light reactions (e.g., through stimulation of NPQ [174]).

Changes in the H^+^-ATP-ase activity and pH accompanying ESs are likely to also participate in the induction of other physiological responses [8]. Potentially, pH changes can affect the induction of expression of defense genes (an artificial inactivation of H^+^-ATP-ase [175] or induction of proton influx [176] activates the genes encoding pin1 and pin2), activation of respiration (modification of the H^+^-ATP-ase activity strongly influences the magnitude of this response, and the artificial inactivation of H^+^-ATP-ase induces a response of respiration which is similar to the ES-induced one [166]), changes in transpiration (modification of the H^+^-ATP-ase activity strongly influences the magnitude of the transpiration response [44]), and suppression of plant growth (the apoplastic alkalization can decrease “acid growth” [177]).

#### 3.2.2. Activation of Ca^2+^ Channels and Increase in Cytoplasmic Concentration of Calcium Ions

Activation of Ca^2+^ channels and an increase in the Ca^2+^ concentration in the cytoplasm are key processes in the induction of AP and VP [8,28,29,30,34,35]; probably, Ca^2+^ channels also participate in SP generation. Considering the great role of calcium signaling in living organisms (including plants [178]), it can be supposed that the Ca^2+^ concentration increase should participate in the induction of physiological responses by ESs.

The participation of calcium signaling in suppression of the phloem mass flow in plants of the Fabaceae family (*Vicia faba*) was investigated in series of works in detail [95,141,142,144]. Representatives of the Fabaceae family contain Ca^2+^-responsive proteins, the forisomes, in their sieve tubes. In the absence of Ca^2+^ ions, the forisomes represent tightly packed spindles anchored at the plasma membrane, while upon entrance of Ca^2+^ into the phloem, they detach from the plasma membrane, disperse, and seal the sieve tubes. The works [95,144] showed that a weak Ca^2+^ influx related to propagation of ESs weakly influences the mass flow (detachment/swelling of forisomes and dispersion of forisome ends are observed), the strong and short-term Ca^2+^ influx induces the short-term suppression of the mass flow (full forisome dispersion), and the strong and long-term Ca^2+^ influx induces the long-term suppression of the mass flow (full forisome dispersion and callose deposition). Ca^2+^-dependent mechanisms also probably participate in the suppression of the phloem mass flow in other plant families (e.g., the effect was shown in *Cucurbita maxima*) [143]. It cannot be fully excluded that this effect of ESs is based on the activity of homologs of forisomes in other plants; however, this suggestion is speculation now.

It is probable that the Ca^2+^ influx participates in forming other physiological responses induced by ESs [8] including expression of defense genes (Ca^2+^ ionophores induce the expression of these genes [179,180], while ES-induced activation of the expression was suppressed at the disruption of the Ca^2+^ influx [181]), an increase in the concentration of stress phytohormones (at least JA, because the Ca^2+^ influx is necessary for the increase in its production induced by ESs [123]; additionally, Ca^2+^ is known as an inductor of ABA [182] and JA [183] synthesis), activation of respiration (application of a Ca^2+^ ionophore induced the respiratory response which was similar to the ES-induced response [138]), and fast photosynthetic inactivation (application of a Ca^2+^ ionophore induced photosynthetic changes similar to ES-induced changes [129], while application of a blocker of Ca^2+^ channels eliminated the photosynthetic response [98]).

#### 3.2.3. Increase in ROS Concentration

Waves of ROS (and, in particular, an increase in H_2_O_2_) are considered as a potential mechanism of propagation of ESs [17,19,39,40]. This means that an increase in ROS concentrations can also be the mechanism of induction of ES-caused physiological responses. It is known that increased ROS concentrations can stimulate the expression of defense genes [184] and ABA [182] and JA [185,186] production. Investigations of ROS waves [186,187,188,189,190] caused by excess light and/or high temperature showed that the waves induce the stimulation of the expression of defense genes and JA production. Additionally, the fast photosynthetic inactivation can also be related to an increase in the ROS concentration because a treatment with an inhibitor of ROS production decreased the response [98].

#### 3.2.4. Increase in ABA and JA Concentrations

An increase in ABA and JA concentrations can be a result of the propagation of ESs [96,97,100,124,125]; however, ES-caused increases in the concentrations of these hormones were shown to be likely inductors of physiological changes in plants [8,34]. It is known that ABA and JA can induce the expression of pin2 genes [11,191] (moreover, disruption of ABA or JA synthesis eliminates the ES-induced increase in the pin2 expression [11,123,125,126]) and cause long-term photosynthetic inactivation [96,97] (photosynthetic parameters are strongly correlated with phytohormone concentrations, and the inactivation is eliminated in ABA-deficient plants) and, probably, the second transpiration decrease [44,96,97,152] (transpiration changes are strongly correlated with the concentrations of both phytohormones and are dependent on modification of the H^+^-ATP-ase activity).

#### 3.2.5. Interactions between Potential Mechanisms of Induction of Physiological Responses

Finally, it should be noted that the mechanisms discussed above can strongly interact [8,11,17,19,39,40]: ROS are considered as a potential wound substance inducing Ca^2+^ influx, and this influx inactivates H^+^-ATP-ase in the plasma membrane and probably stimulates ROS production (through RBOHD); ROS and Ca^2+^ are very likely to stimulate the production of ABA and JA; and ABA and JA can induce ROS, Ca^2+^, and H^+^ signals. This means that the specific roles of each mechanism in ES-induced physiological changes can be weakly distinguished in some cases.

## 4. Electrical Signals and Plant Tolerance to Action of Stressors: Potential Role of PCD

### 4.1. Evidence Supporting Positive Effects of ESs on Plant Tolerance to Stressors

ES-induced physiological changes are considered to increase plant tolerance to the actions of stressors [8,34,40,41,42]. This hypothesis is supported by the following experimental results. (i) Induction of ESs increases whole plant tolerance to low [41] and high [44] temperatures. (ii) Induction of ESs increases the tolerance of the photosynthetic machinery to low [42] and high [42,43,44,45,47] temperatures. The last effect is complex: an ES-induced decrease in PSII damage is observed under increased temperatures [42,47]; however, both an ES-induced decrease in PSI damage and an increase in PSII damage can be observed under high temperatures [43,44,45]. (iii) Induction of ESs stimulates reparation of PSII after the actions of non-optimal temperatures [42,45]. (iv) Generation of local electrical responses in the zone of the action of stressors (gradual temperature increase [46] or decrease [192,193,194]) decreases plant damage; the effect depends on the parameters of electrical responses [46]. (v) Induction of ESs by local actions of heating and excess light (or local irritations without measurements of ESs) eliminates the decrease in the chlorophyll content under the action of heating, and the increase in ion leakage under excess light [39,189]. (vi) Induction of ESs (or local irritations without measurements of ESs) causes expression of defense genes in non-irritated zones of plants [11,101,116,117,118,119,186,187,188,189,190,195,196] (e.g., pin1, pin2, and vsp2 genes protecting against insect attacks [11,101,116,117,118,119], or the ZAT12 gene participating in light acclimation [187]). (vii) Induction of ESs (or local irritations without measurements of ESs) causes increased production of ABA and JA [96,97,100,124,125]; these phytohormones participate in plant tolerance to abiotic and biotic stressors [12,13,197,198,199,200]. Interestingly, ESs can stimulate ethylene production [127] which is also known to participate in the adaptation of plants to stressors [200,201]. (viii) ESs induce physiological responses which are known as adaptive changes under the actions of stressors (e.g., increase in NPQ and stimulation of the cyclic electron flow around PSI [34,129,130,131,132]).

**Figure 1:**
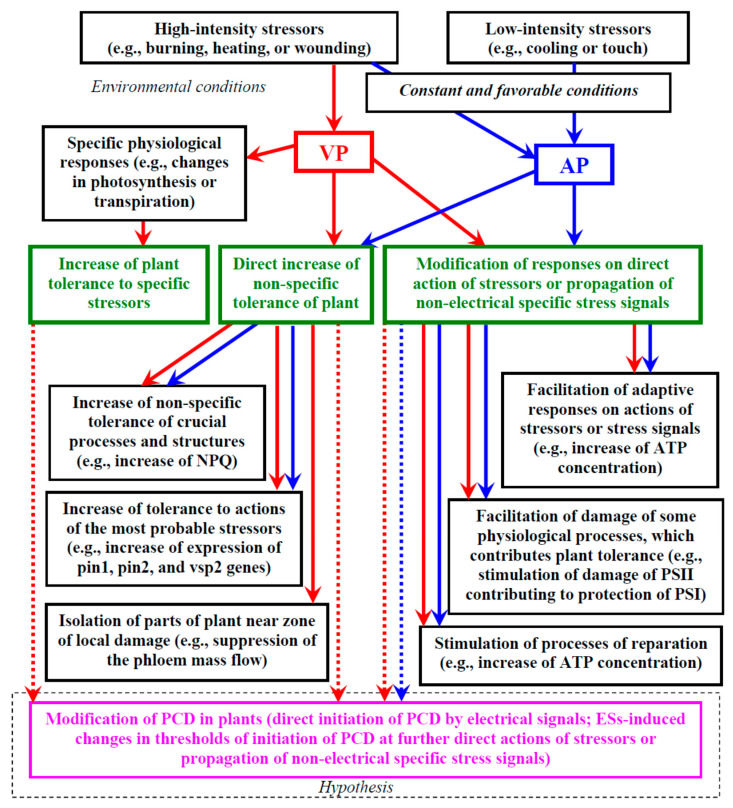
Potential ways of influence of electrical signals on plant tolerance to actions of stressors. Only two types of electrical signals (ESs), variation potential (VP) and action potential (AP), are shown; system potential is not analyzed. Ways related to VP are marked in red; ways related to AP are marked in blue. Dotted lines and box show hypothetical influence of electrical signals on programmed cell death (PCD). For details, see Section 4.

Thus, it is highly probable that there is a positive influence of ESs on plant tolerance to the actions of stressors. Figure 1 shows a brief scheme of the potential ways electrical signals can influence a plant’s tolerance to stressors which are described in more detail below. 

### 4.2. Increase in Plant Tolerance to Specific Stressors Induced by Electrical Signals

The problem of the possibility of a specific influence of ESs on plant tolerance to stressors includes two aspects [8]. First, are there different influences of different types of ESs (mainly AP and VP because SP are weakly investigated signals) on plant tolerance to stressors? Second, can specific signals (AP or VP) encode information about different local stressors, induce different physiological responses, and cause plant tolerance to the action of specific stressors?

#### 4.2.1. Specific Tolerance on Basis of Different Types of Electrical Signals

Our previous analysis [8] showed that it is probable that different types of ESs differently affect plant tolerance; however, a direct experimental comparison between tolerance changes induced by AP vs. VP is absent at present. Briefly, physiological changes induced by AP seem to be similar to the changes induced by VP (e.g., changes in respiration [136,137,138], photosynthesis [128,131,134,137,150,202], production of stress phytohormones [11,202], and expression of genes [117]). However, VP might have additional ways of influencing physiological processes (e.g., the additional mechanism of the stimulation of JA production [126]), and the signal can cause a response in plants in the absence of AP-induced responses (e.g., the fast photosynthetic response in some plant species [112,203] or the suppression of the phloem mass flow [95,141,142,143]).

Considering these points, we hypothesize [8] that the VP influence on physiological processes is stronger than the AP influence that can be related to the longer duration of the VP signal. However, the parameters of VPs (amplitude, duration, shape, and velocity) can depend on the distance from the damaged zone and the intensity of stimuli [8,31,74,99,100,103,104,105]. This means that VP-induced changes in physiological processes and tolerance can be limited by the distance of the VP propagation [8] which, in turn, depends on the intensity of the stressor. Different propagations of VPs caused by burning, heating, or crushing in leaves [99], or different propagations of VP in different leaves [104,135], which are accompanied by different photosynthetic responses, support this suggestion. Thus, the parameters of VP propagation (amplitude, duration, shape, propagation distance, and velocity) can encode the intensities of damages as well as the distance from the zone of their actions [8]; however, these results do not support an ability to encode information about the type of stressor, which is necessary for an increase in the specific tolerance.

In contrast, the self-propagating AP can potentially induce similar physiological responses in the whole plant body [8]; however, the effect should be moderate in comparison with VP-induced changes. Additionally, the properties of APs in higher plants seem to be contradictory (see our previous review [8] for details): (i) potentially, the signal can be induced by weak stimuli (e.g., weak cooling), (ii) AP has the long-term refractory period [80] (in particular, the probability of AP propagation after 1 h of rest is about 50% [8]), and (iii) there are higher plants which have non-propagating AP [8], or AP does not influence their physiological processes [112,203]. Altogether, these points rather support a facultative role of APs in higher plants (however, in algae or mosses, AP can be the key ES). Earlier, we concluded [8] that propagation of AP in higher plants can be observed under stable and favorable environmental conditions; in contrast, fluctuations in the conditions (e.g., fluctuation in the light intensity or mechanical touches) and/or the non-optimal parameters of the conditions (e.g., low or high temperatures) can disturb the plant rest period and limit AP propagation. This can be explained on the basis of the hypothesis by Retivin et al. [41,42] (with our modifications [8]): after a long-term time interval with stable and favorable conditions, even weak changes in environmental conditions can be predictors of future actions of stressors (i.e., they require the systemic physiological response, which is induced by AP); in contrast, under changeable and/or non-optimal conditions, the weak changes can be results of noise in the environmental conditions (i.e., AP propagation and induction of this systemic response are not useful for plants under these conditions).

#### 4.2.2. Induction of Specific Tolerance on Basis of Same Type of Electrical Signal

The following question is still of utmost importance [8]: can electrical signals of a specific type (AP or VP) encode information about the type of stressor and thus increase the specific tolerance of the plant to the actions of this stressor?

Theoretically, the plant AP is not likely to transmit specific information about the stressor [8] because the “all-or-none” law [28] prevents information coding by the amplitude, and the long-term refractory period [80] excludes this coding by the frequency of the AP propagation. Experiments showed that APs induced by different stimuli cause similar responses (see our review [8]), e.g., an electrical current, which directly influences potential-dependent ion channels, and mechanical stimulation, which activates mechanosensitive ion channels (both stimuli are well known as AP-inducing stimuli), caused similar changes in gene expression, ABA and JA production, and photosynthesis [11,125,126,145,202].

Potentially, VP can encode the specific information about stressors because the parameters of the signal (amplitude, duration, shape, propagation distance, and velocity) depend on the type of damage [8,31]. Some experimental works support this suggestion. (i) ESs induced by different chemical agents cause different photosynthetic and transpiration changes in willow [204]. (ii) VPs induced by re-irrigation and by heating, respectively, have different parameters and induce different changes in photosynthesis and stomata conductance in maize [151]. (iii) Burning- and heating-induced VPs propagate into pea leaves [99]; in contrast, a crush-induced VP does not propagate into the leaves. The fast photosynthetic inactivation is observed at the propagation of burning- and heating-induced VPs, but it is absent at the propagation of the crush-induced VP [99]. (iv) Burning- and heating-induced VPs have different amplitudes and durations; they cause different changes in the apoplastic pH, parameters of photosynthesis and transpiration, and concentration of ABA, JA, and SA in wheat leaves [100]. (v) ESs induced by light with different spectral bands cause different plant tolerances to biotic damages: thus, systemic signals induced by white light were shown to increase plant tolerance to a phytopathogen 1 h after induction, the signals induced by red light increased this tolerance at 8 h, and the signals induced by blue light were influenced 24 h after induction [36,205]. (vi) An ROS wave induced by the local action of excess light decreases the stomata aperture in non-irritated leaves of Arabidopsis; in contrast, an ROS wave induced by the local heating increases the aperture [189]. Considering the proposed relations between the ROS waves and ESs (see, e.g., [17,19,39,40]), these results additionally support the possibility that a VP encodes information about the type of stressor. Moreover, combinations of actions of excess light and heating eliminate the changes in the stomatal aperture [189].

These results support the possibility of VPs to encode information about the type of stressor. This encoding could be the basis of induction of the increase in the plant tolerance to the action of specific stressors. There is work [189] which experimentally supports this increase at the propagation of ROS waves. It shows that a light-induced ROS wave increases the tolerance of non-irritated leaves to the excess light; a heating-induced ROS wave does not influence the tolerance. In contrast, the heating-induced ROS wave increases the tolerance of non-irritated leaves to the increased temperature; the light-induced ROS wave does not influence the tolerance. The tolerance changes are based on the accumulation of many different stress-specific transcripts and metabolites [189]; the accumulation differs at the propagation of ROS waves induced by the local action of the excess light and heating.

The last result supports the possibility of the VP-induced increase in the plant tolerance to the action of specific stressors. However, this problem requires further investigations because some points are not fully clear (Can ROS waves be considered as the signal which is identical to VP? Can similar effects be formed in other plants?).

### 4.3. Direct Increase in Non-Specific Plant Tolerance Induced by Electrical Signals

In accordance with the hypothesis by Retivin et al. [41,42], ESs can increase the non-specific plant tolerance to stressors. The hypothesis was initially proposed for APs in higher plants [41]; however, considering the similarity of physiological responses induced by AP and VP, we hypothesized [8] a similar influence of AP and VP on the non-specific plant tolerance to the actions of stressors takes place in plants. The positive influence of both signals on plant tolerance [41,42,44,45,46,47] supports this hypothesis. Therefore, we will use the general term “electrical signals” (without division of APs and VPs) in the following sections. 

There are two general directions of increase in the non-specific tolerance [8]: a direct increase in the tolerance before the actions of stressors, and a modification of plant responses to the direct action of stressors or propagation of other non-electrical specific stress signals. The first direction is analyzed in this section.

We suppose that several potential pathways can be used for the direct increase in the non-specific plant tolerance induced by ESs: (i) increase in the non-specific tolerance of crucial processes and structures, (ii) increase in the tolerance to actions of the most probable stressors, and (iii) isolation of parts of the plant near the zone of local damage.

#### 4.3.1. Increase in Non-Specific Tolerance of Crucial Processes and Structures

There are some processes and structures which can be damaged by the actions of different types of stressors; their damages are extremely dangerous for the plant organism. In particular, the light-induced damage of the photosynthetic machinery, which is crucial for plant life, can also be stimulated by other types of stressors (e.g., drought or non-optimal temperatures) [8,206,207,208]. The photosynthetic damages stimulate the overproduction of ROS that can disrupt other structures in cells. Considering these points, preliminary protection of the photosynthetic machinery and minimization of the photodamage can increase plant tolerance to further actions of different stressors (i.e., the non-specific plant tolerance) [8,34]. It is known that ESs induce photosynthetic changes, which participate in the decrease in photodamage [34]: increase in NPQ [34,129,130,131,133] (including the energy-dependent component of NPQ [133]) and stimulation of the cyclic electron flow around PSI [132]. It can be expected that the photosynthetic changes should directly increase the non-specific tolerance of the photosynthetic machinery to stressors. A similar physiological role can also be assigned to ROS wave-induced (i.e., possibly ES-induced) activation of expression of the ZAT12 gene which participates in light acclimation [187].

The plasma membrane is another important target of actions of different types of stressors [209] as supported by the increase in ion leakage under the action of water deficit [210], excess light [189], and high temperatures [210]. It is known [192,193,194] that depolarization of the membrane potential and K^+^ efflux contribute to protection of the plasma membrane. Considering the strong depolarization [31,34,35] and the increase in the apoplastic K^+^ concentration [90], which accompany the generation of ESs, these mechanisms can also participate in the increase in the non-specific plant tolerance to stressors [8].

#### 4.3.2. Increase in Tolerance to Actions of the Most Probable Stressors

Another pathway of direct increase in plant tolerance can be based on the non-specific activation of specific mechanisms decreasing damage under the action of the most probable stressors [8]. In particular, ESs which are induced by abiotic stressors (burning, mechanical wounding, or electrical current) stimulate expression of pin1, pin2, and vsp2 [11,101,116,117,118,119] or suppress the phloem mass flow [95,141,142,143,144,145] that disturbs insect feeding. These responses cannot be characterized as specific because in these examples, abiotic stressors induce protection against insect attacks; however, these non-specific responses can contribute to plant survival at the relatively high probability of biotic damage.

Potentially, a decrease in transpiration [44,152,156] caused by the local burning and, probably, ES propagation can play a similar role. It is known that the magnitude of the decrease is the largest under a low relative water content in air [152]. The probability of water deficit seems to be high under such conditions; this means that the stomata closing induced by the non-specific local action of stressors can also contribute to plant survival. 

The ES-induced increase in ABA (protecting against water deficit) and JA (protecting against biotic damages) is in good accordance with both mechanisms (stimulation of expression of defense genes and suppression of transpiration) [8].

#### 4.3.3. Isolation of Parts of Plant near Zone of Local Damage

This mechanism is mainly based on the VP-induced suppression of the phloem mass flow [95,141,142,143,144,145] which can isolate part of the conductive system of the plant; an AP does not induce this effect. The latter result seems to be expected because an AP is the self-propagating signal [28]. This means that a hypothetical AP-induced suppression of the mass flow would be observed in the whole plant body and should damage the plant. In contrast, VP is expected to induce the suppression within a specific distance from the damaged zone (the VP amplitude and duration are decreased with an increase in distance from the damaged zone [104,135], and the mass flow suppression depends on the magnitude and duration of the increase in the Ca^2+^ concentration [95,144] related to the VP parameters).

The isolation can be considered [8,95,144] as a protective process; at least, a decrease in the phloem mass flow should restrict the propagation of pathogens and uncontrolled propagation of strongly disturbed concentrations of ions (e.g., shifted pH or increased Ca^2+^ concentration) and neutral molecules (e.g., strongly increased ROS concentrations) from the damaged zone. Additionally, a decrease in the phloem mass flow contributes to an increase in concentrations of sugars in the cells near the damaged zone [8,34] that can, therefore, also play a protective role (e.g., as a source of energy, see below).

Thus, VP-induced isolation of part of the conductive system of the plant can be considered as the extremal protective response which can be induced before the direct action of the stressor. 

### 4.4. Modification of Responses on Direct Action of Stressors or Propagation of Other Specific Stress Signals

Modification of plant physiological responses to the direct action of stressors or propagation of non-electrical specific stress signals can represent another effective direction of increase in the whole plant tolerance to adverse factors [8]. There are some potential pathways contributing to this effect [8,34]: (i) facilitating adaptive responses to direct actions of stressors or further propagation of other stress signals, (ii) facilitating damage of specific processes by stressors, which contributes to the whole plant tolerance, and (iii) stimulation of reparation processes.

#### 4.4.1. Facilitating Adaptive Responses to Direct Actions of Stressors or Propagation of other Stress Signals

It can be supposed [8,34] that the propagation of ESs facilitates the formation of adaptive responses induced by the further direct action of stressors or further propagation of non-electrical specific stress signals. This facilitation should decrease the plant damage under the action of stressors due to specific changes in protection against specific stressors; i.e., it increases the whole plant tolerance.

The ES-induced increase in the ATP content in plants [34,135,211] can be considered as a mechanism of facilitating the physiological responses induced by the direct action of stressors or specific stress signals because ATP is necessary for the most of these responses (e.g., synthesis of protective proteins) [8]. This increase is based on the ES-induced activation of respiration [130,131,135,136,137,138], the suppression of the mesophyll CO_2_ conductance, the decrease in photosynthetic dark reactions [34,128,130,131,132], and, probably, the decrease in the sugar outflow by phloem unloading [139,140] and the suppression of the phloem mass flow [95,141,142,143,144,145].

Potentially, modifications of the NPQ induction, which is an important pathway of adaptation of the photosynthetic machinery to actions of stressors [174,206,212,213,214,215,216], can also be the mechanism facilitating adaptive responses. It has been shown that ESs induce the long-term stimulation of the transition from violaxanthin to zeaxanthin [202]; these transitions can be considered as “light memory” which accelerates NPQ forming under repeated light action [174,216]. Probably, an increase in the zeaxanthin concentration can also accelerate NPQ stimulation under the actions of other stressors.

An alternative variant of the ES influence is the stimulation of the formation of adaptive responses induced by non-electrical specific stress signals, which are propagated through the plant body [36,39,40]. In accordance with [39], ES-induced increased concentrations of serine and sucrose can stimulate respiration and photorespiration (the activation of respiration is observed after propagation of ESs [130,131,135,136,137,138]) which contribute to an increase in NAD(P)H concentrations in mesophyll cells. These processes can facilitate the formation of adaptive responses induced by both the direct actions of stressors and non-electrical specific stress signals [39,40].

Finally, ES effects on the expression of numerous adaptive genes including genes encoding components of signaling cascades [36,39,40,101,187,188,189,190,205] can also contribute to the adaptive responses induced by the direct actions of stressors and non-electrical specific stress signals (e.g., a moderate increase in concentrations of transcripts of the signal pathway can facilitate the induction of adaptive responses related to this pathway).

#### 4.4.2. The Protective Role of Facilitating Damage of some Physiological Processes by Stressors and Stimulation of Repair

The damage of physiological processes can play a positive role in plant tolerance under high-intensity actions of stressors [8]. For example, damage of PSII, which can be repaired within relatively short-term time intervals (hours), can protect PSI that contributes to the whole tolerance of the photosynthetic machinery (reparation of PSI is a long-term process, and damage of PSI disrupts ATP synthesis, which is related to the cyclic electron flow around PSI) [34,217,218,219,220]. Additionally, a decrease in the photosynthetic electron flow which is caused by PSII damage [8,219,220] can lower ROS production and thereby, probably, the ROS-induced disruption of other physiological processes. This means that stimulation of PSII damage (e.g., facilitating its damage by stressors) can be the extremal pathway of protection of the photosynthetic machinery and the whole plant.

It is known [34,43,44,45] that ESs can facilitate heating-induced damage of PSII (in particular, through stimulation of leaf heating under high temperatures [44]). This increase in PSII damage is accompanied by an increase in PSI thermotolerance [43] and a decrease in heat-induced suppression of plant growth [44]. Importantly, this effect is only observed under high temperatures [43]; under moderate heating, ESs protect PSII against the heat damage [47]. These results suggest [34] that ESs can stimulate the stress tolerance of plants through facilitation of the damage of some physiological processes (e.g., photosynthetic processes).

Stimulation of repair processes is another potential target of ESs [8,34]. In particular, it is known [42,45] that induction of ESs can stimulate processes of reparation of the photosynthetic machinery damaged by some stressors, for instance, by non-optimal temperatures. It can be supposed [8,34] that this effect is related to the ES-induced increase in the ATP concentration in plants [135,211] because the increased ATP levels contribute to reparation of the photosynthetic machinery after the actions of stressors (e.g., increased temperatures or excess light) [206,221]. Potentially, this mechanism can also contribute to the ES-induced increase in the reparation of other processes impaired due to the action of stressors [8].

### 4.5. Potential Pathways of Induction of Programmed Cell Death in Plants by Electrical Signals

PCD is a process of controlled elimination of specific cells in living organisms (including plants) [59,222,223] which includes several components: apoptosis, relatively slow autophagic PCD, and regulated necrosis. In animals, apoptosis is characterized by the shrinkage of cells, condensation of chromatin, and destruction of the nucleus [59]; it is caused by cytochrome release from the mitochondria and activation of caspases and endonucleases [59]. “Apoptotic-like cell death”, which has some similar properties, occurs in plants. Autophagic PCD [72], which is widely observed in plants, is based on activation of autophagic proteins, appearance of autophagosomes, their fusion with vacuoles, stimulation of the vacuolar processing enzymes, and, finally, destruction of vacuoles. Regulated necrosis includes the types of PCD which cannot be characterized as apoptosis (apoptotic-like cell death) or autophagic PCD [59,223], e.g., ferroptosis, which is based on stimulation of lipid peroxidation [223].

**Figure 2:**
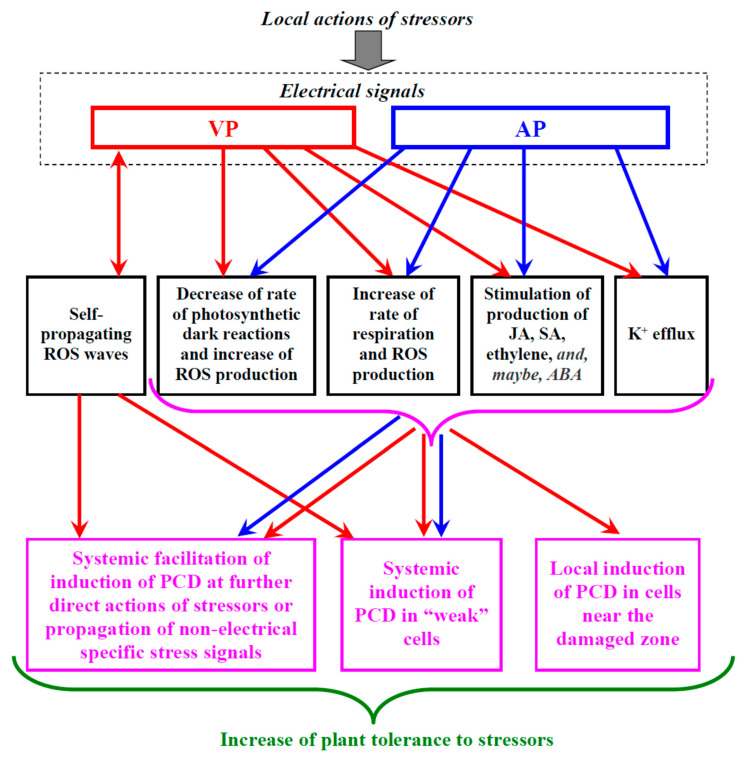
Potential pathways of influence of electrical signals on induction of programmed cell death (PCD). Only two types of electrical signals (VP and AP) are shown; system potential is not analyzed. Pathways related to VP are marked in red; pathways related to AP are marked in blue. For details, see Section 4.5 and Section 4.6.

PCD can participate in organism development (dPCD) and in responses to the action of environmental stressors (ePCD) [59,61,223]. It can be expected that just ePCD can be affected by ESs participating in the systemic plant responses to stressor actions [8]. In our review, we analyze the possibility of the ES influence on the PCD induction in plants and focus on the most general factors of this induction; analysis of PCD details is beyond the scope of this review. An increase in the ROS concentration (e.g., hydrogen peroxide) is widely considered as a “universal” factor of PCD induction [59,60,61,62,63,223]. This means that there are, at least, several potential pathways of PCD induction by ESs (Figure 2).

#### 4.5.1. ROS Waves

ROS waves are considered as one of the potential mechanisms of VP (see Section 2.4). RBOHD in the plasma membrane catalyzes the secondary generation of a superoxide radical [17,19,31] which is quickly reduced to hydrogen peroxide participating in long-distance signaling. Production of the superoxide radical is localized on the outer side of the membrane; this means that its participation in the induction of PCD is unlikely (its lifetime is about 1 µs [61] which is not enough for the transport of the superoxide radical through the plasma membrane). In contrast, H_2_O_2_ is a relatively long-lived molecule (its lifetime ranging from 1 ms to several seconds [61,64]) which can be transported from the apoplast to the cytoplasm through aquaporins located in the plasma membrane, PIP2 [19]. An increase in the hydrogen peroxide concentration is a widely known mechanism of induction of PCD [60,61], and RBOHD participates in the regulation of PCD including its stimulation near the damaged zone (phytopathogens) [224].

Thus, ROS waves, particularly H_2_O_2_ waves, can potentially participate in PCD induction; this hypothesis is supported by long-term increase of ROS content after propagation of stress signals [195]. This hypothesis implies that VP (but not AP) can induce PCD via ROS waves. However, ROS waves cannot be considered as the only mechanism of VP propagation. The properties of self-propagating ROS waves [19,40] are not in accordance with some properties of VP propagation (see Section 2.4). This means that several mechanisms of VP propagation are possible; i.e., results which are shown in investigations of ROS waves cannot be relevant for other pathways of VP propagation, e.g., the hydraulic mechanism [8,25,31,35,111].

#### 4.5.2. Decrease in the Rate of Photosynthetic Dark Reactions and Increase in the Rate of Respiration

Fast and long-term decreases in the rate of photosynthetic dark reactions caused by a decrease in the mesophyll CO_2_ conductance [34,128] are typical photosynthetic responses induced by ESs [8,34,130,131,132,134]. Overreduction of the electron transport chain in chloroplasts is probably a result of the ES-induced photosynthetic inactivation [34,130]. Increased production of singlet oxygen, a superoxide radical, and hydrogen peroxide can be caused by this overreduction [34,62,225]. Potentially, the increased ROS production can induce PCD because H_2_O_2_ can directly cause the response [60,61], while singlet oxygen [62,225,226] and the superoxide radical [225] can modify JA and SA synthesis, which can also cause PCD. This pathway is supported by studies which show an important role of chloroplasts and the activity of their electron transport chain in the initiation of PCD [227,228].

Investigation of light-induced long-distance stress signals in Arabidopsis, which are probably electrical signals [36,205], showed that the signals decrease the stomata conductance and induce overreduction of the plastoquinone pool [229]; these responses were accompanied by stimulation of PCD. The authors supposed [205] that the limitations of the CO_2_ flux into the leaf due to stomata closure suppress photosynthetic dark reactions and stimulate photorespiration that increase H_2_O_2_ production and induce PCD. These results are in good accordance with the stimulation of PCD under an artificial decrease in stomata conductance and restriction of the CO_2_ flux into the leaf [230]. It is interesting that PCD stimulation can be observed even under a 50% decrease in the stomata conductance [229,230]. Considering the high magnitudes of ES-induced decreases in mesophyll CO_2_ conductance [128] and stomata conductance [44,131], induction of PCD by this pathway seems likely.

ES-induced stimulation of respiration [130,131,135,136,137,138], which should increase the ROS production, seems to represent an additional potential mechanism of PCD induction because mitochondria are considered to be an important source of ROS for initiation of PCD [231,232].

It should be noted that these proposed mechanisms can both directly induce PCD and indirectly stimulate PCD initiation under the continuing actions of stressors or during propagation of non-electrical specific stress signals. An ES-induced increase in the ATP concentration [135,211] can also stimulate the induction of the responses because energization of plant cells can contribute to the induction of PCD under the action of stressors (the action of stressors on low-energized cells rather induces necrosis) [233].

#### 4.5.3. Stimulation of Production of Stress Phytohormones

As discussed above, ESs can stimulate the production of several stress phytohormones including JA [11,96,97,100,101,123,124,125], SA [100], ethylene [127], and ABA [11,96,97,100,124]. The increase can be long term: its duration equals about several hours or more (e.g., strongly increased concentrations of ABA are observed 5–6 h after the local stimulation of plants and induction of ESs [126,202]).

JA [61,66,225,234], SA [66,224,234], and ethylene [66,224,234] are considered to participate in the induction of PCD in plants (particularly through stimulation of ROS production). Investigation of light-induced long-distance stress signals in Arabidopsis [229] (probably ESs [36,205]) showed that ethylene participates in PCD induction for 1 h after the signal initiation; JA and SA additionally stimulate PCD after long time intervals (several hours). Results have shown that ESs can potentially stimulate PCD via increased JA, SA, and ethylene production. It cannot be excluded that ABA, which induces stomata closure, decreases mesophyll CO_2_ conductance, and suppresses photosynthetic dark reactions [165,169] (i.e., induces ROS production), can also stimulate PCD in plants [61,235,236].

#### 4.5.4. K^+^ Efflux

Generation of electrical signals accompanies changes in the activity of H^+^-ATP-ase, Ca^2+^, anions, and inward-rectifying and outward-rectifying K^+^ channels in the plasma membrane [8,31,34,35,237]. These processes cause large and often long-term changes in the concentrations of protons, calcium ions, chlorine ions, and potassium ions [90,134,150,164]; the changes, in turn, participate in the initiation of ES-induced physiological responses [8,34,166]. In particular, it has been shown that changes in K^+^ concentrations in the cytoplasm (decrease) and apoplast (increase) can equal several mM or more [8,90]; at VP generation, the K^+^ efflux can be increased near the damaged zone [31].

K^+^ leakage, which is stimulated by stressors, leads to a decrease in the cytoplasmic pool of potassium ions and is considered as a mechanism of PCD induction through the activation of caspase-like proteases by potassium ions [68,69,70,71,72,73]. The leakage is related to ROS activation of K^+^ permeable non-selective cation channels (NSCC) [69]. However, the actions of many stressors (e.g., cooling or salt stress) can also induce depolarization of the plasma membrane [8,209] that inactivates Arabidopsis K^+^ transporters (AKT) and activates guard cell outward-rectifying K^+^ channels (GORK) [69]. It is known that depolarization is the necessary initial stage of AP and VP generations [8,31,34,35]; moreover, activation of GORK channels plays an important role in the generation of electrical signals [91]. This means that the generation of ESs can potentially modify the induction of PCD through an increase in the K^+^ efflux.

However, the induction of PCD requires large changes in the cytoplasmic K^+^ concentration (e.g., about 50 mM [71]); in contrast, a moderate decrease in the cytoplasmic K^+^ concentration can stimulate catabolic processes and thereby help in saving “metabolic” energy for adaptation and repair processes [70,72,73]. This means that direct AP-induced initiation of PCD is rather unlikely; in contrast, it is probable that VP can directly induce PCD near the damaged zone because their magnitude is maximal in this plant part (and, thereby, changes in K^+^ are also maximal). Additionally, the increased concentration of K^+^ after ES generation should facilitate PCD induction under further direct actions of stressors or during propagation of the non-electrical stress signal.

### 4.6. Potential Roles of the ES-Induced Programmed Cell Death in Increase in Plant Tolerance to Stressors

Section 4.5 shows that there are indirect arguments supporting the participation of ESs which are caused by the local actions of stressors in induction of ePCD: in particular, ESs strongly influence processes which can induce PCD. However, we have only few direct pieces of experimental evidence supporting the ES effect on PCD (e.g., [229]). As a result, we can only speculate about the role of the ES-induced stimulation of PCD in the increase in plant tolerance to stressors. Below, we consider some potential possibilities (Figure 2).

#### 4.6.1. Local PCD Induction in Cells near the Damaged Zone

ES-induced PCD can participate in the elimination of plant cells near the damaged zone, which is observed after the action of local damages on plants [224]. It can be supposed that this response protects plants by means of restriction of the damaged zone (e.g., restriction of phytopathogen propagations on the basis of elimination of potential “targets” for infection). However, this potential pathway strongly requires ESs which are attenuated with an increase in the distance from the damaged zone (i.e., VP [8,31,74,99,100,103,104] which weaken with the increasing distance). The magnitudes of physiological changes induced by these signals should also be attenuated with the increasing distance from the damaged zone and weakening of the signals because they depend on the parameters of ESs [8]. This means that these magnitudes should be enough for PCD initiation only within the specific distance from the damaged zone. 

In contrast, signals with constant amplitudes (e.g., self-propagating AP [8,28,35] or ROS waves [19,40,99]) will likely induce similar physiological changes over the whole distance from the zone of stimulation. This means that these signals would induce PCD in the whole plant body provided that the magnitudes of ES-induced changes suffice for the PCD initiation or would not induce the response provided that the magnitudes of ES-induced changes do not suffice for the PCD initiation. Thus, these signals (AP and ROS waves) cannot participate in the PCD initiation in cells near the damaged zone. 

It is interesting that the potential zone of PCD induction by VP (as well as the induction of other physiological responses [8]) should correlate with the intensity of the action of the local stressor and thus would probably lead to a stronger restriction of the damaged zone at more intensive damage.

It should be noted that the described mechanism should modify only damages caused by the actions of local stressors which induce ESs. The influence of PCD induction around the zone of the local damage on the systemic plant tolerance seems to be limited because it is possible for the further direct action of stressors to continue in other parts of the plant which can be far from the zone of the initial local damage. Thus, other potential pathways of the influence of the ES-induced increase in PCD on the systemic plant tolerance should be discussed.

#### 4.6.2. Systemic PCD Induction in “Weak” Cells

ES-induced physiological changes [8,34] can be additional factors inducing PCD: in healthy cells, they are unlikely to induce PCD; however, these changes can initiate PCD in cells which had been weakly or moderately damaged by the stressors (“weak” cells). Regarding this, PCD is not initiated in these cells without the additional influence of ESs. Potentially, the mechanism can exclude the weak cells before the intensive phase of the action of a stressor (as discussed above, ESs can be a sufficient predictor of this action in accordance with this hypothesis [8,41,42]) and, thereby, increase the probability of whole plant survival.

This hypothesis is in good accordance with the positive influence of ESs on systemic plant tolerance [8,34,36,40,41,42,189,205] and seems to be very promising. In particular, the hypothesis supports different actions of attenuated and non-attenuated (self-propagating) electrical signals on PCD initiation (and plant tolerance): self-propagating signals should induce similar changes in the whole plant body (possibly induction of PCD in moderately stressed plant cells); in contrast, the magnitude of changes induced by attenuated signals should be decreased with the increase in the distance from the damaged zone (e.g., induction of PCD in weakly and moderately stressed cells near the zone of the local stressor action, and this induction in moderately stressed cells further from this zone). It is probable that this systemic induction of PCD can also participate in the ES-induced increase in plant tolerance to specific stressors, which was shown in some works [189].

However, there are experimental results which rather contradict this chain of events (local damage—ES propagation—elimination of weakly/moderately stressed cells in the plant body—increase in the systemic tolerance to the further intensive action of the stressor on the whole plant or a large part of it). It is important that the proposed mechanism can also positively influence the plant tolerance at the induction of electrical signals after initiation of the direct action of the stressor (as the additional regulatory mechanism). This probability has been weakly investigated; however, work [205] showed that the induction of ESs after initiation of a phytopathogen infection does not positively influence plant damage. Thus, this potential pathway of participation of the ES-induced PCD stimulation in plant tolerance requires further investigations.

#### 4.6.3. Systemic Facilitation of PCD Induction at Further Direct Actions of Stressors and/or Non-Electrical Specific Stress Signals

The next pathway of influence of the ES-caused stimulation of PCD on plant tolerance can be based on the facilitation of the induction of programmed cell death at the further direct actions of stressors on the whole plant body (or a large part of it) and/or propagation of non-electrical specific stress signals. This means that only changes induced by ESs prior to the action of stressors or only further changes induced by the direct stressor action or by propagation of specific stress signals cannot induce PCD in this case; in contrast, a combined action of both processes induces programmed cell death. Alternatively, ES-induced changes can accelerate the induction of PCD at the further direct action of the stressor or during propagation of the specific stress signal.

This pathway is also in good agreement with the ES-induced increase in the non-specific and/or specific plant tolerance to the actions of stressors [8,34,36,40,41,42,189,205]. Potentially, it seems to be the most effective of all the proposed mechanisms because the facilitation of PCD induction by stressors does not strongly influence PCD in plant cells without the direct action of stressors and/or the propagation of specific stress signals; i.e., the plant does not spend its resources in this case. In contrast, if the direct action of a specific stressor or the propagation of a specific stress signal require the initiation of PCD in specific plant cells (e.g., cells at the early stages of damage), then preliminary induction of ESs and physiological changes (such as propagation of ROS waves [17,31,39,40], a decrease in photosynthetic dark reactions [8,34,128,130,131,132,134], activation of respiration [130,131,135,136,137,138], synthesis of stress phytohormones [11,96,97,100,101,123,124,125,127], and/or K^+^ leakage [8,31,90]) should facilitate this initiation and increase the probability of plant survival. Like the induction of PCD in “weak” cells (see above), this mechanism can potentially provide dependence of the PCD response on the intensity of a stressor action and the distance from the damaged zone and support specific changes in PCD (at least for VPs).

The experimental work of [205] additionally supports this pathway of PCD regulation: the induction of ESs can ameliorate further plant damage by phytopathogens, and participation of changes in PCD in this response is very likely. Other works [41,44] which showed an increase in plant tolerance to stressors (non-optimal temperatures) after induction of ESs are also in accordance with this pathway of activation of PCD. Finally, it should be noted that ES-induced facilitation of PCD initiation by further actions of stressors or propagation of specific signals resembles other mechanisms of increase in plant tolerance (Figure 1, [8,34]) through facilitation of adaptive processes (e.g., by means of an increase in the ATP contents [135,211]).

## 5. Conclusions and Perspectives

Our review shows that the generation and propagation of electrical signals (particularly variation potentials and action potentials) can be important mechanisms of an increase in plant tolerance to the actions of abiotic and biotic stressors. This increase probably includes non-specific and specific components which support plant survival in environmental conditions. Initiation of programmed cell death by electrical signals seems to be probable in plants. There are several potential pathways relating ES-induced physiological responses to PCD, which include ROS waves, a decreased rate of photosynthetic dark reactions, activation of respiration, synthesis of stress phytohormones, and K^+^ leakage. Potentially, these mechanisms can influence plant tolerance through local PCD initiation near the damaged zone, systemic PCD activation in “weak” cells, and systemic facilitation of PCD initiation at further direct actions of stressors and/or propagation of non-electrical specific stress signals.

Finally, several perspectives of the investigations of the relationship between electrical signals and PCD can be proposed: (i) direct analysis of the possibility of PCD initiation by electrical signals which are induced by different stressors and/or in different plant species; (ii) comparison between influences of variation potentials and action potentials on PCD; (iii) analysis of influence of electrical signals (possibly induced by different stimuli) on PCD initiation at further direct actions of stressors (e.g., excess light or non-optimal temperatures); (iv) analysis of the role of changes in PCD induced by electrical signals in modifications of plant tolerance to different stressors; (v) quantification and simulation of investigated processes that can be used for the complex theoretical analysis of relations between electrical signals, plant tolerance to stressors, and PCD at different conditions. We suppose that the solution to these problems will provide a basis of revealing and characterizing novel mechanisms of regulation of plant tolerance by electrical signals.

## Data Availability

No new data were created or analyzed in this study. Data sharing is not applicable to this article.

## References

[1] Dempsey D.A., Klessig D.F. (2012). SOS—Too many signals for systemic acquired resistance?. Trends Plant Sci..

[2] Shah J., Zeier J. (2013). Long-distance communication and signal amplification in systemic acquired resistance. Front. Plant Sci..

[3] Huber A.E., Bauerle T.L. (2016). Long-distance plant signaling pathways in response to multiple stressors: The gap in knowledge. J. Exp. Bot..

[4] Devireddy A.R., Zandalinas S.I., Gómez-Cadenas A., Blumwald E., Mittler R. (2018). Coordinating the overall stomatal response of plants: Rapid leaf-to-leaf communication during light stress. Sci. Signal..

[5] Hilleary R., Gilroy S. (2018). Systemic signaling in response to wounding and pathogens. Curr. Opin. Plant Biol..

[6] Suzuki N., Katano K. (2018). Coordination between ROS regulatory systems and other pathways under heat stress and pathogen attack. Front. Plant Sci..

[7] Takahashi F., Shinozaki K. (2019). Long-distance signaling in plant stress response. Curr. Opin. Plant. Biol..

[8] Sukhov V., Sukhova E., Vodeneev V. (2019). Long-distance electrical signals as a link between the local action of stressors and the systemic physiological responses in higher plants. Progr. Biophys. Mol. Biol..

[9] Qiu X.M., Sun Y.Y., Ye X.Y., Li Z.G. (2020). Signaling role of glutamate in plants. Front. Plant Sci..

[10] Farmer E.E., Gao Y.Q., Lenzoni G., Wolfender J.L., Wu Q. (2020). Wound- and mechanostimulated electrical signals control hormone responses. New Phytol..

[11] Peña-Cortés H., Fisahn J., Willmitzer L. (1995). Signals involved in wound-induced proteinase inhibitor II gene expression in tomato and potato plants. Proc. Natl. Acad. Sci. USA.

[12] Sreenivasulu N., Harshavardhan V.T., Govind G., Seiler C., Kohli A. (2012). Contrapuntal role of ABA: Does it mediate stress tolerance or plant growth retardation under long-term drought stress?. Gene.

[13] de Ollas C., Dodd I.C. (2016). Physiological impacts of ABA–JA interactions under water-limitation. Plant Mol. Biol..

[14] Takahashi F., Hanada K., Kondo T., Shinozaki K. (2019). Hormone-like peptides and small coding genes in plant stress signaling and development. Curr. Opin. Plant. Biol..

[15] Toyota M., Spencer D., Sawai-Toyota S., Jiaqi W., Zhang T., Koo A.J., Howe G.A., Gilroy S. (2018). Glutamate triggers long-distance, calcium-based plant defense signaling. Science.

[16] Slesak I., Libik M., Karpinska B., Karpinski S., Miszalski Z. (2007). The role of hydrogen peroxide in regulation of plant metabolism and cellular signaling in response to environmental stresses. Acta Biochim. Pol..

[17] Suzuki N., Mittler R. (2012). Reactive oxygen species-dependent wound responses in animals and plants. Free Radic. Biol. Med..

[18] Gaupels F., Durner J., Kogel K.H. (2017). Production, amplification and systemic propagation of redox messengers in plants? The phloem can do it all!. New Phytol..

[19] Fichman Y., Myers R.J., Grant D.G., Mittler R. (2021). Plasmodesmata-localized proteins and ROS orchestrate light-induced rapid systemic signaling in Arabidopsis. Sci. Signal..

[20] Choi W.G., Toyota M., Kim S.H., Hilleary R., Gilroy S. (2014). Salt stress-induced Ca^2+^ waves are associated with rapid, long-distance root-to-shoot signaling in plants. Proc. Natl. Acad. Sci. USA.

[21] Xiong T.C., Ronzier E., Sanchez F., Corratgé-Faillie C., Mazars C., Thibaud J.B. (2014). Imaging long distance propagating calcium signals in intact plant leaves with the BRET-based GFP-aequorin reporter. Front. Plant Sci..

[22] Kiep V., Vadassery J., Lattke J., Maaß J.P., Boland W., Peiter E., Mithöfer A. (2015). Systemic cytosolic Ca^2+^ elevation is activated upon wounding and herbivory in Arabidopsis. New Phytol..

[23] Storti M., Costa A., Golin S., Zottini M., Morosinotto T., Alboresi A. (2018). Systemic calcium wave propagation in *Physcomitrella patens*. Plant Cell Physiol..

[24] Malone M. (1994). Wound-induced hydraulic signals and stimulus transmission in *Mimosa pudica* L. New Phytol..

[25] Mancuso S. (1999). Hydraulic and electrical transmission of wound-induced signals in *Vitis vinifera*. Aust. J. Plant Physiol..

[26] Christmann A., Weiler E.W., Steudle E., Grill E. (2007). A hydraulic signal in root-to-shoot signalling of water shortage. Plant J..

[27] Christmann A., Grill E., Huang J. (2013). Hydraulic signals in long-distance signaling. Curr. Opin. Plant Biol..

[28] Trebacz K., Dziubinska H., Krol E., Baluška F., Mancuso S., Volkmann D. (2006). Electrical signals in long-distance communication in plants. Communication in Plants. Neuronal Aspects of Plant Life.

[29] Fromm J., Lautner S. (2007). Electrical signals and their physiological significance in plants. Plant Cell Environ..

[30] Gallé A., Lautner S., Flexas J., Fromm J. (2015). Environmental stimuli and physiological responses: The current view on electrical signaling. Environ. Exp. Bot..

[31] Vodeneev V., Akinchits E., Sukhov V. (2015). Variation potential in higher plants: Mechanisms of generation and propagation. Plant Signal. Behav..

[32] Choi W.G., Hilleary R., Swanson S.J., Kim S.H., Gilroy S. (2016). Rapid, long-distance electrical and calcium signaling in plants. Annu. Rev. Plant Biol..

[33] Hedrich R., Salvador-Recatalà V., Dreyer I. (2016). Electrical wiring and long-distance plant communication. Trends Plant Sci..

[34] Sukhov V. (2016). Electrical signals as mechanism of photosynthesis regulation in plants. Photosynth. Res..

[35] Sukhova E., Akinchits E., Sukhov V. (2017). Mathematical models of electrical activity in plants. J. Membr. Biol..

[36] Szechyńska-Hebda M., Lewandowska M., Karpiński S. (2017). Electrical signaling, photosynthesis and systemic acquired acclimation. Front. Physiol..

[37] Vega-Muñoz I., Duran-Flores D., Fernández-Fernández Á.D., Heyman J., Ritter A., Stael S. (2020). Breaking bad news: Dynamic molecular mechanisms of wound response in plants. Front. Plant Sci..

[38] Lang R.D., Volkov A.G. (2008). Solitary waves in soybean induced by localized thermal stress. Plant Signal. Behav..

[39] Suzuki N., Miller G., Salazar C., Mondal H.A., Shulaev E., Cortes D.F., Shuman J.L., Luo X., Shah J., Schlauch K. (2013). Temporal-spatial interaction between reactive oxygen species and abscisic acid regulates rapid systemic acclimation in plants. Plant Cell..

[40] Choi W.G., Miller G., Wallace I., Harper J., Mittler R., Gilroy S. (2017). Orchestrating rapid long-distance signaling in plants with Ca^2+^, ROS and electrical signals. Plant J..

[41] Retivin V.G., Opritov V.A., Fedulina S.B. (1997). Generation of action potential induces preadaptation of Cucurbita pepo L. stem tissues to freezing injury. Russ. J. Plant Physiol..

[42] Retivin V.G., Opritov V.A., Lobov S.A., Tarakanov S.A., Khudyakov V.A. (1999). Changes in the resistance of photosynthesizing cotyledon cells of pumpkin seedlings to cooling and heating, as induced by the stimulation of the root system with KCl solution. Russ. J. Plant Physiol..

[43] Sukhov V., Surova L., Sherstneva O., Vodeneev V. (2014). Influence of variation potential on resistance of the photosynthetic machinery to heating in pea. Physiol. Plant..

[44] Sukhov V., Surova L., Sherstneva O., Bushueva A., Vodeneev V. (2015). Variation potential induces decreased PSI damage and increased PSII damage under high external temperatures in pea. Funct. Plant. Biol..

[45] Surova L., Sherstneva O., Vodeneev V., Sukhov V. (2016). Variation potential propagation decreases heat-related damage of pea photosystem I by 2 different pathways. Plant Signal. Behav..

[46] Sukhov V., Gaspirovich V., Mysyagin S., Vodeneev V. (2017). High-temperature tolerance of photosynthesis can be linked to local electrical responses in leaves of pea. Front. Physiol..

[47] Grinberg M.A., Gudkov S.V., Balalaeva I.V., Gromova E., Sinitsyna Y., Sukhov V., Vodeneev V. (2021). Effect of chronic β-radiation on long-distance electrical signals in wheat and their role in adaptation to heat stress. Environ. Exp. Bot..

[48] Chatterjee S.K., Ghosh S., Das S., Manzella V., Vitaletti A., Masi E., Santopolo L., Mancuso S., Maharatna K. (2014). Forward and inverse modelling approaches for prediction of light stimulus from electrophysiological response in plants. Measurement.

[49] Chatterjee S.K., Das S., Maharatna K., Masi E., Santopolo L., Mancuso S., Vitaletti A. (2015). Exploring strategies for classification of external stimuli using statistical features of the plant electrical response. J. R. Soc. Interface.

[50] Chen Y., Zhao D.-J., Wang Z.-Y., Wang Z.-Y., Tang G., Huang L. (2016). Plant electrical signal classification based on waveform similarity. Algorithms.

[51] Souza G.M., Ferreira A.S., Saraiva G.F., Toledo G.R. (2017). Plant “electrome” can be pushed toward a self-organized critical state by external cues: Evidences from a study with soybean seedlings subject to different environmental conditions. Plant Signal. Behav..

[52] Saraiva G.F.R., Ferreira A.S., Souza G.M. (2017). Osmotic stress decreases complexity underlying the electrophysiological dynamic in soybean. Plant Biol..

[53] Chatterjee S.K., Malik O., Gupta S. (2018). Chemical sensing employing plant electrical signal response-classification of stimuli using curve fitting coefficients as features. Biosensors.

[54] Debono M.W., Souza G.M. (2019). Plants as electromic plastic interfaces: A mesological approach. Prog. Biophys. Mol. Biol..

[55] Qin X.-H., Wang Z.-Y., Yao J.-P., Zhou Q., Zhao P.-F., Wang Z.-Y., Huang L. (2020). Using a one-dimensional convolutional neural network with a conditional generative adversarial network to classify plant electrical signals. Comp. Electron. Agric..

[56] Simmi F.Z., Dallagnol L.J., Ferreira A.S., Pereira D.R., Souza G.M. (2020). Electrome alterations in a plant-pathogen system: Toward early diagnosis. Bioelectrochemistry.

[57] Parise A.G., Reissig G.N., Basso L.F., Senko L.G.S., Oliveira T.F.C., de Toledo G.R.A., Ferreira A.S., Souza G.M. (2021). Detection of different hosts from a distance alters the behaviour and bioelectrical activity of *Cuscuta racemosa*. Front. Plant Sci..

[58] Elmore S. (2007). Apoptosis: A review of programmed cell death. Toxicol. Pathol..

[59] Locato V., De Gara L. (2018). Programmed cell death in plants: An overview. Methods Mol. Biol..

[60] Gechev T.S., Hille J. (2005). Hydrogen peroxide as a signal controlling plant programmed cell death. J. Cell Biol..

[61] Gadjev I., Stone J.M., Gechev T.S. (2008). Programmed cell death in plants: New insights into redox regulation and the role of hydrogen peroxide. Int. Rev. Cell Mol. Biol..

[62] Fischer B.B., Hideg É., Krieger-Liszkay A. (2013). Production, detection, and signaling of singlet oxygen in photosynthetic organisms. Antioxid. Redox Signal..

[63] Petrov V., Hille J., Mueller-Roeber B., Gechev T.S. (2015). ROS-mediated abiotic stress-induced programmed cell death in plants. Front. Plant Sci..

[64] Smirnoff N., Arnaud D. (2019). Hydrogen peroxide metabolism and functions in plants. New Phytol..

[65] Love A.J., Milner J.J., Sadanandom A. (2008). Timing is everything: Regulatory overlap in plant cell death. Trends Plant Sci..

[66] Reinbothe C., Springer A., Samol I., Reinbothe S. (2009). Plant oxylipins: Role of jasmonic acid during programmed cell death, defence and leaf senescence. FEBS J..

[67] Mira M., Hill R.D., Stasolla C. (2016). Regulation of programmed cell death by phytoglobins. J. Exp. Bot..

[68] Peters J., Chin C.K. (2007). Potassium loss is involved in tobacco cell death induced by palmitoleic acid and ceramide. Arch. Biochem. Biophys..

[69] Anschütz U., Becker D., Shabala S. (2014). Going beyond nutrition: Regulation of potassium homoeostasis as a common denominator of plant adaptive responses to environment. J. Plant Physiol..

[70] Demidchik V., Straltsova D., Medvedev S.S., Pozhvanov G.A., Sokolik A., Yurin V. (2014). Stress-induced electrolyte leakage: The role of K^+^-permeable channels and involvement in programmed cell death and metabolic adjustment. J. Exp. Bot..

[71] Shabala S., Pottosin I. (2014). Regulation of potassium transport in plants under hostile conditions: Implications for abiotic and biotic stress tolerance. Physiol. Plant..

[72] Demidchik V., Tyutereva E.V., Voitsekhovskaja O.V. (2018). The role of ion disequilibrium in induction of root cell death and autophagy by environmental stresses. Funct. Plant Biol..

[73] Demidchik V. (2019). ROS-activated ion channels in plants: Biophysical characteristics, physiological functions and molecular nature. Int. J. Mol. Sci..

[74] Stahlberg R., Cleland R.E., van Volkenburgh E., Baluška F., Mancuso S., Volkmann D. (2006). Slow wave potentials - A propagating electrical signal unique to higher plants. Communication in Plants. Neuronal Aspects of Plant Life.

[75] Zimmermann M.R., Maischak H., Mithöfer A., Boland W., Felle H.H. (2009). System potentials, a novel electrical long-distance apoplastic signal in plants, induced by wounding. Plant. Physiol..

[76] Zimmermann M.R., Mithöfer A., Will T., Felle H.H., Furch A.C. (2016). Herbivore-triggered electrophysiological reactions: Candidates for systemic signals in higher plants and the challenge of their identification. Plant Physiol..

[77] Sibaoka T. (1991). Rapid plant movements triggered by action potentials. Bot. Mag. Tokyo..

[78] Van Bel A.J., Knoblauch M., Furch A.C., Hafke J.B. (2011). (Questions)(n) on phloem biology. 1. Electropotential waves, Ca^2+^ fluxes and cellular cascades along the propagation pathway. Plant Sci..

[79] Krol E., Dziubinska H., Stolarz M., Trebacz K. (2006). Effects of ion channel inhibitors on cold- and electrically-induced action potentials in Dionaea muscipula. Biol. Plant..

[80] Favre P., Degli Agosti R. (2007). Voltage-dependent action potentials in *Arabidopsis thaliana*. Physiol. Plant..

[81] Kisnieriene V., Lapeikaite I., Sevriukova O., Ruksenas O. (2016). The effects of Ni^2+^ on electrical signaling of *Nitellopsis obtusa* cells. J. Plant Res..

[82] Kisnieriene V., Lapeikaite I., Pupkis V. (2018). Electrical signalling in *Nitellopsis obtusa*: Potential biomarkers of biologically active compounds. Funct. Plant Biol..

[83] Koselski M., Pupkis V., Hashimoto K., Lapeikaite I., Hanaka A., Wasko P., Plukaite E., Kuchitsu K., Kisnieriene V., Trebacz K. (2021). Impact of mammalian two-pore channel inhibitors on long-distance electrical signals in the characean macroalga *Nitellopsis obtusa* and the early terrestrial liverwort *Marchantia polymorpha*. Plants.

[84] Krol E., Dziubińska H., Trebacz K. (2004). Low-temperature-induced transmembrane potential changes in mesophyll cells of *Arabidopsis thaliana*, *Helianthus annuus* and *Vicia faba*. Physiol. Plant..

[85] Degli Agosti R. (2014). Touch-induced action potentials in Arabidopsis thaliana. Arch. Des. Sci..

[86] Krausko M., Perutka Z., Šebela M., Šamajová O., Šamaj J., Novák O., Pavlovič A. (2017). The role of electrical and jasmonate signalling in the recognition of captured prey in the carnivorous sundew plant *Drosera capensis*. New Phytol..

[87] Pavlovič A., Jakšová J., Novák O. (2017). Triggering a false alarm: Wounding mimics prey capture in the carnivorous Venus flytrap (*Dionaea muscipula*). New Phytol..

[88] Trebacz K., Sievers A. (1998). Action potentials evoked by light in traps of *Dionaea muscipula* Ellis. Plant Cell Physiol..

[89] Pikulenko M.M., Bulychev A.A. (2005). Light-triggered action potentials and changes in quantum efficiency of photosystem II in Anthoceros cells. Russ. J. Plant Physiol..

[90] Felle H.H., Zimmermann M.R. (2007). Systemic signalling in barley through action potentials. Planta.

[91] Cuin T.A., Dreyer I., Michard E. (2018). The role of potassium channels in Arabidopsis thaliana long distance electrical signalling: AKT2 modulates tissue excitability while GORK shapes action potentials. Int. J. Mol. Sci..

[92] Vodeneev V.A., Opritov V.A., Pyatygin S.S. (2006). Reversible changes of extracellular pH during action potential generation in a higher plant *Cucurbita pepo*. Russ. J. Plant Physiol..

[93] Sukhov V., Vodeneev V. (2009). A mathematical model of action potential in cells of vascular plants. J. Membr. Biol..

[94] Zhao D.J., Chen Y., Wang Z.Y., Xue L., Mao T.L., Liu Y.M., Wang Z.Y., Huang L. (2015). High-resolution non-contact measurement of the electrical activity of plants in situ using optical recording. Sci. Rep..

[95] van Bel A.J., Furch A.C., Will T., Buxa S.V., Musetti R., Hafke J.B. (2014). Spread the news: Systemic dissemination and local impact of Ca^2+^ signals along the phloem pathway. J. Exp. Bot..

[96] Hlavácková V., Krchnák P., Naus J., Novák O., Spundová M., Strnad M. (2006). Electrical and chemical signals involved in short-term systemic photosynthetic responses of tobacco plants to local burning. Planta.

[97] Hlavinka J., Nožková-Hlaváčková V., Floková K., Novák O., Nauš J. (2012). Jasmonic acid accumulation and systemic photosynthetic and electrical changes in locally burned wild type tomato, ABA-deficient sitiens mutants and sitiens pre-treated by ABA. Plant Physiol. Biochem..

[98] Białasek M., Górecka M., Mittler R., Karpiński S. (2017). Evidence for the Involvement of electrical, calcium and ROS signaling in the systemic regulation of non-photochemical quenching and photosynthesis. Plant Cell Physiol..

[99] Vodeneev V., Mudrilov M., Akinchits E., Balalaeva I., Sukhov V. (2018). Parameters of electrical signals and photosynthetic responses induced by them in pea seedlings depend on the nature of stimulus. Funct. Plant Biol..

[100] Mudrilov M., Ladeynova M., Berezina E., Grinberg M., Brilkina A., Sukhov V., Vodeneev V. (2021). Mechanisms of specific systemic response in wheat plants under different locally acting heat stimuli. J. Plant Physiol..

[101] Mousavi S.A., Chauvin A., Pascaud F., Kellenberger S., Farmer E.E. (2013). GLUTAMATE RECEPTOR-LIKE genes mediate leaf-to-leaf wound signalling. Nature.

[102] Salvador-Recatalà V., Tjallingii W.F., Farmer E.E. (2014). Real-time, in vivo intracellular recordings of caterpillar-induced depolarization waves in sieve elements using aphid electrodes. New Phytol..

[103] Stahlberg R., Cosgrove D.J. (1997). The propagation of slow wave potentials in pea epicotyls. Plant Physiol..

[104] Sukhova E., Mudrilov M., Vodeneev V., Sukhov V. (2018). Influence of the variation potential on photosynthetic flows of light energy and electrons in pea. Photosynth. Res..

[105] Sukhov V., Akinchits E., Katicheva L., Vodeneev V. (2013). Simulation of variation potential in higher plant cells. J. Membr. Biol..

[106] Stahlberg R., Cosgrove D.J. (1996). Induction and ionic basis of slow wave potentials in seedlings of *Pisum sativum* L. Planta.

[107] Katicheva L., Sukhov V., Bushueva A., Vodeneev V. (2015). Evaluation of the open time of calcium channels at variation potential generation in wheat leaf cells. Plant Signal. Behav..

[108] Evans M.J., Morris R.J. (2017). Chemical agents transported by xylem mass flow propagate variation potentials. Plant J..

[109] Blyth M.G., Morris R.J. (2019). Shear-enhanced dispersion of a wound substance as a candidate mechanism for variation potential transmission. Front. Plant Sci..

[110] Vodeneev V., Orlova A., Morozova E., Orlova L., Akinchits E., Orlova O., Sukhov V. (2012). The mechanism of propagation of variation potentials in wheat leaves. J. Plant Physiol..

[111] Sukhova E., Akinchits E., Gudkov S.V., Pishchalnikov R.Y., Vodeneev V., Sukhov V. (2021). A Theoretical analysis of relations between pressure changes along xylem vessels and propagation of variation potential in higher plants. Plants.

[112] Lautner S., Grams T.E.E., Matyssek R., Fromm J. (2005). Characteristics of Electrical Signals in Poplar and Responses in Photosynthesis. Plant Physiol..

[113] Sibaoka T. (1969). Physiology of rapid movements in higher plants. Ann. Rev. Plant Physiol..

[114] Volkov A.G., Adesina T., Markin V.S., Jovanov E. (2008). Kinetics and mechanism of *Dionaea muscipula* trap closing. Plant Physiol..

[115] Hedrich R., Neher E. (2018). Venus flytrap: How an excitable, carnivorous plant works. Trends Plant Sci..

[116] Wildon D.C., Thain J.F., Minchin P.E.H., Gubb I.R., Reilly A.J., Skipper Y.D., Doherty H.M., O’Donnell P.J., Bowles D. (1992). Electrical signalling and systemic proteinase inhibitor Induction in the wounded plant. Nature.

[117] Stanković B., Davies E. (1996). Both action potentials and variation potentials induce proteinase inhibitor gene expression in tomato. FEBS Lett..

[118] Stanković B., Davies E. (1997). Intercellular communication in plants: Electrical stimulation of proteinase inhibitor gene expression in tomato. Planta.

[119] Davies E., Vian A., Vian C., Stankovic B. (1997). Rapid systemic up-regulation of genes after heat-wounding and electrical stimulation. Acta Physiol. Plant..

[120] Vian A., Henry-Vian C., Schantz R., Ledoigt G., Frachisse J.M., Desbiez M.O., Julien J.L. (1996). Is membrane potential involved in calmodulin gene expression after external stimulation in plants?. FEBS Lett..

[121] Stankovic B., Davies E. (1998). The wound response in tomato involves rapid growth and electrical responses, systemically up-regulated transcription of proteinase inhibitor and calmodulin and down-regulated translation. Plant Cell Physiol..

[122] Vian A., Henry-Vian C., Davies E. (1999). Rapid and systemic accumulation of chloroplast mrna-binding protein transcripts after flame stimulus in tomato. Plant Physiol..

[123] Fisahn J., Herde O., Willmitzer L., Peña-Cortés H. (2004). Analysis of the transient increase in cytosolic Ca^2+^ during the action potential of higher plants with high temporal resolution: Requirement of Ca^2+^ transients for induction of jasmonic acid biosynthesis and PINII gene expression. Plant Cell Physiol..

[124] Ladeynova M., Mudrilov M., Berezina E., Kior D., Grinberg M., Brilkina A., Sukhov V., Vodeneev V. (2020). Spatial and temporal dynamics of electrical and photosynthetic activity and the content of phytohormones induced by local stimulation of pea plants. Plants.

[125] Herde O., Atzorn R., Fisahn J., Wasternack C., Willmitzer L., Pena-Cortes H. (1996). Localized wounding by heat initiates the accumulation of proteinase inhibitor II in abscisic acid-deficient plants by triggering jasmonic acid biosynthesis. Plant Physiol..

[126] Herde O., Peña Cortés H., Wasternack C., Willmitzer L., Fisahn J. (1999). Electric signaling and pin2 gene expression on different abiotic stimuli depend on a distinct threshold level of endogenous abscisic acid in several abscisic acid-deficient tomato mutants. Plant Physiol..

[127] Dziubinska H., Filek M., Koscielniak J., Trebacz K. (2003). Variation and action potentials evoked by thermal stimuli accompany enhancement of ethylene emission in distant non-stimulated leaves of *Vicia faba* minor seedlings. J. Plant Physiol..

[128] Gallé A., Lautner S., Flexas J., Ribas-Carbo M., Hanson D., Roesgen J., Fromm J. (2013). Photosynthetic responses of soybean (*Glycine max* L.) to heat-induced electrical signalling are predominantly governed by modifications of mesophyll conductance for CO_2_. Plant Cell Environ..

[129] Krupenina N.A., Bulychev A.A. (2007). Action potential in a plant cell lowers the light requirement for non-photochemical energy-dependent quenching of chlorophyll fluorescence. Biochim. Biophys. Acta.

[130] Pavlovič A., Slováková L., Pandolfi C., Mancuso S. (2011). On the mechanism underlying photosynthetic limitation upon trigger hair irritation in the carnivorous plant Venus flytrap (*Dionaea muscipula* Ellis). J. Exp. Bot..

[131] Sukhov V., Orlova L., Mysyagin S., Sinitsina J., Vodeneev V. (2012). Analysis of the photosynthetic response induced by variation potential in geranium. Planta.

[132] Sukhov V., Surova L., Sherstneva O., Katicheva L., Vodeneev V. (2015). Variation potential influence on photosynthetic cyclic electron flow in pea. Front. Plant Sci..

[133] Sukhov V., Sukhova E., Gromova E., Surova L., Nerush V., Vodeneev V. (2019). The electrical signal-induced systemic photosynthetic response is accompanied by changes in the photochemical reflectance index in pea. Func. Plant Biol..

[134] Sukhov V., Sherstneva O., Surova L., Katicheva L., Vodeneev V. (2014). Proton cellular influx as a probable mechanism of variation potential influence on photosynthesis in pea. Plant Cell Environ..

[135] Surova L., Sherstneva O., Vodeneev V., Katicheva L., Semina M., Sukhov V. (2016). Variation potential-induced photosynthetic and respiratory changes increase ATP content in pea leaves. J. Plant Physiol..

[136] Filek M., Kościelniak J. (1997). The effect of wounding the roots by high temperature on the respiration rate of the shoot and propagation of electric signal in horse bean seedlings (*Vicia faba* L. minor). Plant Sci..

[137] Lautner S., Stummer M., Matyssek R., Fromm J., Grams T.E.E. (2014). Involvement of respiratory processes in the transient knockout of net CO_2_ uptake in *Mimosa pudi*ca upon heat stimulation. Plant Cell Environ..

[138] Khlopkov A., Sherstneva O., Ladeynova M., Grinberg M., Yudina L., Sukhov V., Vodeneev V. (2021). Participation of calcium ions in induction of respiratory response caused by variation potential in pea seedlings. Plant Signal. Behav..

[139] Fromm J. (1991). Control of phloem unloading by action potentials in Mimosa. Physiol. Plant..

[140] Fromm J., Bauer T. (1994). Action potentials in maize sieve tubes change phloem translocation. J. Exp. Bot..

[141] Furch A.C., Hafke J.B., Schulz A., van Bel A.J. (2007). Ca^2+^-mediated remote control of reversible sieve tube occlusion in *Vicia faba*. J. Exp. Bot..

[142] Furch A.C., van Bel A.J., Fricker M.D., Felle H.H., Fuchs M., Hafke J.B. (2009). Sieve element Ca^2+^ channels as relay stations between remote stimuli and sieve tube occlusion in *Vicia faba*. Plant Cell..

[143] Furch A.C., Zimmermann M.R., Will T., Hafke J.B., van Bel A.J. (2010). Remote-controlled stop of phloem mass flow by biphasic occlusion in *Cucurbita maxima*. J. Exp. Bot..

[144] Hafke J.B., Furch A.C.U., Fricker M.D., van Bel A.J.E. (2009). Forisome dispersion in Vicia faba is triggered by Ca2+ hotspots created by concerted action of diverse Ca^2+^ channels in sieve elements. Plant Signal. Behav..

[145] Herde O., Fuss H., Peña-Cortés H., Fisahn J. (1995). Proteinase inhibitor II gene expression induced by electrical stimulation and control of photosynthetic activity in tomato plants. Plant Cell Physiol..

[146] Fromm J., Fei H. (1998). Electrical signaling and gas exchange in maize plants of drying soil. Plant Sci..

[147] Koziolek C., Grams T.E.E., Schreiber U., Matyssek R., Fromm J. (2004). Transient knockout of photosynthesis mediated by electrical signals. New Phytol..

[148] Kaiser H., Grams T.E. (2006). Rapid hydropassive opening and subsequent active stomatal closure follow heat-induced electrical signals in *Mimosa pudica*. J. Exp. Bot..

[149] Grams T.E., Koziolek C., Lautner S., Matyssek R., Fromm J. (2007). Distinct roles of electric and hydraulic signals on the reaction of leaf gas exchange upon re-irrigation in *Zea mays* L. Plant Cell Environ..

[150] Grams T.E., Lautner S., Felle H.H., Matyssek R., Fromm J. (2009). Heat-induced electrical signals affect cytoplasmic and apoplastic pH as well as photosynthesis during propagation through the maize leaf. Plant Cell Environ..

[151] Vuralhan-Eckert J., Lautner S., Fromm J. (2018). Effect of simultaneously induced environmental stimuli on electrical signalling and gas exchange in maize plants. J. Plant Physiol..

[152] Yudina L.M., Sherstneva O.N., Mysyagin S.A., Vodeneev V.A., Sukhov V.S. (2019). Impact of local damage on transpiration of pea leaves at various air humidity. Russ. J. Plant Physiol..

[153] Shiina T., Tazawa M. (1986). Action potential in *Luffa cylindrica* and its effects on elongation growth. Plant Cell Physiol..

[154] Sukhova E., Yudina L., Akinchits E., Vodeneev V., Sukhov V. (2019). Influence of electrical signals on pea leaf reflectance in the 400-800-nm range. Plant Signal. Behav..

[155] Sukhova E., Yudina L., Gromova E., Nerush V., Vodeneev V., Sukhov V. (2020). Burning-induced electrical signals influence broadband reflectance indices and water index in pea leaves. Plant Signal. Behav..

[156] Sukhova E., Yudina L., Gromova E., Ryabkova A., Vodeneev V., Sukhov V. (2021). Influence of local burning on difference reflectance indices based on 400-700 nm wavelengths in leaves of pea seedlings. Plants.

[157] Gamon J.A., Peñuelas J., Field C.B. (1992). A narrow-waveband spectral index that tracks diurnal changes in photosynthetic efficiency. Remote Sens. Environ..

[158] Garbulsky M.F., Peñuelas J., Gamon J., Inoue Y., Filella I. (2011). The photochemical reflectance index (PRI) and the remote sensing of leaf, canopy and ecosystem radiation use efficiencies. A review and meta-analysis. Remote Sens. Environ..

[159] Zhang C., Filella I., Garbulsky M.F., Peñuelas J. (2016). Affecting factors and recent improvements of the photochemical reflectance index (PRI) for remotely sensing foliar, canopy and ecosystemic radiation-use efficiencies. Remote Sens..

[160] Sukhova E., Sukhov V. (2018). Connection of the photochemical reflectance index (PRI) with the photosystem II quantum yield and nonphotochemical quenching can be dependent on variations of photosynthetic parameters among investigated plants: A meta-analysis. Remote Sens..

[161] Sukhova E., Sukhov V. (2019). Analysis of light-induced changes in the photochemical reflectance index (PRI) in leaves of pea, wheat, and pumpkin using pulses of green-yellow measuring light. Remote Sens..

[162] Sukhova E., Sukhov V. (2020). Relation of photochemical reflectance indices based on different wavelengths to the parameters of light reactions in photosystems I and II in pea plants. Remote Sens..

[163] Peñuelas J., Piñol J., Ogaya R., Filella I. (1997). Estimation of plant water concentration by the reflectance Water Index WI (R900/R970). Int. J. Remote Sens..

[164] Zimmermann M.R., Felle H.H. (2009). Dissection of heat-induced systemic signals: Superiority of ion fluxes to voltage changes in substomatal cavities. Planta.

[165] Yudina L., Sukhova E., Sherstneva O., Grinberg M., Ladeynova M., Vodeneev V., Sukhov V. (2020). Exogenous abscisic acid can influence photosynthetic processes in peas through a decrease in activity of H^+^-ATP-ase in the plasma membrane. Biology.

[166] Yudina L., Sherstneva O., Sukhova E., Grinberg M., Mysyagin S., Vodeneev V., Sukhov V. (2020). Inactivation of H^+^-ATPase participates in the influence of variation potential on photosynthesis and respiration in peas. Plants.

[167] Sherstneva O.N., Surova L.M., Vodeneev V.A., Plotnikova Y.I., Bushueva A.V., Sukhov V.S. (2016). The role of the intra- and extracellular protons in the photosynthetic response induced by the variation potential in pea seedlings. Biochem. Mosc. Suppl. Ser. A Membr. Cell Biol..

[168] Sherstneva O.N., Vodeneev V.A., Surova L.M., Novikova E.M., Sukhov V.S. (2016). Application of a mathematical model of variation potential for analysis of its influence on photosynthesis in higher plants. Biochem. Mosc. Suppl. Ser. A.

[169] Sukhov V.S., Gaspirovich V.V., Gromova E.N., Ladeynova M.M., Sinitsyna Y.V., Berezina E.V., Akinchits E.K., Vodeneev V.A. (2017). Decrease of mesophyll conductance to CO_2_ is a possible mechanism of abscisic acid influence on photosynthesis in seedlings of pea and wheat. Biochem. Mosc. Suppl. Ser. A.

[170] Sukhova E.M., Sukhov V.S. (2018). Dependence of the CO_2_ uptake in a plant cell on the plasma membrane H^+^-ATPase activity: Theoretical analysis. Biochem. Mosc. Suppl. Ser. A Membr. Cell Biol..

[171] Sukhov V., Surova L., Morozova E., Sherstneva O., Vodeneev V. (2016). Changes in H^+^-ATP synthase activity, proton electrochemical gradient, and pH in pea chloroplast can be connected with variation potential. Front Plant Sci..

[172] Sherstneva O.N., Vodeneev V.A., Katicheva L.A., Surova L.M., Sukhov V.S. (2015). Participation of intracellular and extracellular pH changes in photosynthetic response development induced by variation potential in pumpkin seedlings. Biochemistry (Moscow).

[173] Bulychev A.A., Alova A.V., Rubin A.B. (2013). Fluorescence transients in chloroplasts of *Chara corallina* cells during transmission of photoinduced signal with the streaming cytoplasm. Russ. J. Plant Physiol..

[174] Ruban A.V. (2016). Nonphotochemical Chlorophyll fluorescence quenching: Mechanism and effectiveness in protecting plants from photodamage. Plant Physiol..

[175] Schaller A., Oecking C. (1999). Modulation of plasma membrane H^+^-ATPase activity differentially activates wound and pathogen defense responses in tomato plants. Plant Cell..

[176] Schaller A., Frasson D. (2001). Induction of wound response gene expression in tomato leaves by ionophores. Planta.

[177] Geilfus C.M. (2017). The pH of the apoplast: Dynamic factor with functional impact under stress. Mol. Plant..

[178] Kudla J., Becker D., Grill E., Hedrich R., Hippler M., Kummer U., Parniske M., Romeis T., Schumacher K. (2018). Advances and current challenges in calcium signaling. New Phytol..

[179] Takezawa D. (1999). Elicitor- and A23187-induced expression of WCK-1, a gene encoding mitogen-activated protein kinase in wheat. Plant Mol. Biol..

[180] Dombrowski J.E., Bergey D.R. (2007). Calcium ions enhance systemin activity and play an integral role in the wound response. Plant Sci..

[181] Vian A., Henry-Vian C., Schantz R., Schantz M.-L., Davies E., Ledoigt G., Desbiez M.-O. (1997). Effect of calcium and calcium-counteracting drugs on the response of *Bidens pilosa* L. to wounding. Cell Physiol..

[182] Xiong L., Zhu J.-K. (2003). Regulation of abscisic acid biosynthesis. Plant Physiol..

[183] Wang X., Zhu B., Jiang Z., Wang S. (2019). Calcium-mediation of jasmonate biosynthesis and signaling in plants. Plant Sci..

[184] Orozco-Cárdenas M.L., Narváez-Vásquez J., Ryan C.A. (2001). Hydrogen peroxide acts as a second messenger for the induction of defense genes in tomato plants in response to wounding, systemin, and methyl jasmonate. Plant Cell..

[185] Hu X., Li W., Chen Q., Yang Y. (2009). Early signal transduction linking the synthesis of jasmonic acid in plant. Plant Signal. Behav..

[186] Hu X., Neill S.J., Yang Y., Cai W. (2009). Fungal elicitor Pep-25 increases cytosolic calcium ions, H_2_O_2_ production and activates the octadecanoid pathway in *Arabidopsis thaliana*. Planta.

[187] Zandalinas S.I., Fichman Y., Mittler R. (2020). Vascular bundles mediate systemic reactive oxygen signaling during light stress. Plant Cell..

[188] Fichman Y., Zandalinas S.I., Sengupta S., Burks D., Myers R.J., Azad R.K., Mittler R. (2020). MYB30 Orchestrates systemic reactive oxygen signaling and plant acclimation. Plant Physiol..

[189] Zandalinas S.I., Fichman Y., Devireddy A.R., Sengupta S., Azad R.K., Mittler R. (2020). Systemic signaling during abiotic stress combination in plants. Proc. Natl. Acad. Sci. USA.

[190] Choudhury F.K., Devireddy A.R., Azad R.K., Shulaev V., Mittler R. (2018). Local and systemic metabolic responses during light-induced rapid systemic signaling. Plant Physiol..

[191] Hildmann T., Ebneth M., Peña-Cortés H., Sánchez-Serrano J.J., Willmitzer L., Prat S. (1992). General roles of abscisic and jasmonic acids in gene activation as a result of mechanical wounding. Plant Cell..

[192] Opritov V.A., Pyatygin S.S., Krauz V.O. (1993). Role of electrical activity in cooling-induced development of adaptation syndrome in higher plant cells. Russ. J. Plant Physiol..

[193] Opritov V.A., Pyatygin S.S., Krauz V.O., Khudyakov V.A., Abramova N.N. (1994). Activation of the electrogenic plasmalemma H^+^-pump in the adaptation of higher plants to moderate low-temperature stress. Russ. J. Plant Physiol..

[194] Pyatygin S.S., Opritov V.A., Krauz V.O., Polovinkin A.V. (1996). Increase in cold resistance of electrogenesis as a basis for adaptive repolarization in higher plant cells during chilling. Russ. J. Plant Physiol..

[195] Fichman Y., Mittler R. (2021). A systemic whole-plant change in redox levels accompanies the rapid systemic response to wounding. Plant. Physiol..

[196] Suzuki N., Devireddy A.R., Inupakutika M.A., Baxter A., Miller G., Song L., Shulaev E., Azad R.K., Shulaev V., Mittler R. (2015). Ultra-fast alterations in mRNA levels uncover multiple players in light stress acclimation in plants. Plant J..

[197] Hirayama T., Shinozaki K. (2010). Research on plant abiotic stress responses in the post-genome era: Past, present and future. Plant J..

[198] Song S., Qi T., Wasternack C., Xie D. (2014). Jasmonate signaling and crosstalk with gibberellin and ethylene. Curr. Opin. Plant Biol..

[199] Delgado C., Mora-Poblete F., Ahmar S., Chen J.T., Figueroa C.R. (2021). Jasmonates and plant salt stress: Molecular players, physiological effects, and improving tolerance by using genome-associated tools. Int. J. Mol. Sci..

[200] Skalak J., Nicolas K.L., Vankova R., Hejatko J. (2021). Signal integration in plant abiotic stress responses via multistep phosphorelay signaling. Front. Plant Sci..

[201] Riyazuddin R., Verma R., Singh K., Nisha N., Keisham M., Bhati K.K., Kim S.T., Gupta R. (2020). Ethylene: A master regulator of salinity stress tolerance in plants. Biomolecules.

[202] Herde O., Peña-Cortés H., Fuss H., Willmitzer L., Fisahn J. (1999). Effects of mechanical wounding, current application and heat treatment on chlorophyll fluorescence and pigment composition in tomato plants. Physiol. Plant..

[203] Fromm J., Hajirezaei M.R., Becker V.K., Lautner S. (2013). Electrical signaling along the phloem and its physiological responses in the maize leaf. Front. Plant Sci..

[204] Fromm J., Eschrich W. (1993). Electric signals released from roots of willow (*Salix viminalis* L.) change transpiration and photosynthesis. J. Plant Physiol..

[205] Szechyńska-Hebda M., Kruk J., Górecka M., Karpińska B., Karpiński S. (2010). Evidence for light wavelength-specific photoelectrophysiological signaling and memory of excess light episodes in Arabidopsis. Plant Cell..

[206] Allakhverdiev S.I., Kreslavski V.D., Klimov V.V., Los D.A., Carpentier R., Mohanty P. (2008). Heat stress: An overview of molecular responses in photosynthesis. Photosynth. Res..

[207] Roach T., Krieger-Liszkay A. (2014). Regulation of photosynthetic electron transport and photoinhibition. Curr. Protein Pept. Sci..

[208] Derks A., Schaven K., Bruce D. (2015). Diverse mechanisms for photoprotection in photosynthesis. Dynamic regulation of photosystem II excitation in response to rapid environmental change. Biochim. Biophys Acta.

[209] Pyatygin S.S. (2004). Role of plasma membrane in cold action perception in plant cells. Biol. Membr. Mosc..

[210] Ancillotti C., Bogani P., Biricolti S., Calistri E., Checchini L., Ciofi L., Gonnelli C., Del Bubba M. (2015). Changes in polyphenol and sugar concentrations in wild type and genetically modified *Nicotiana langsdorffii* Weinmann in response to water and heat stress. Plant Physiol. Biochem..

[211] Retivin V.G., Opritov V.A., Abramova N.N., Lobov S.A., Fedulina S.B. (1999). ATP level in the phloem exudate of higher plant shoot after propagation of electric responses to the burning or cooling. Vestn. NNSU Ser. Biol..

[212] Demmig-Adams B., Adams W.W. (1996). The role of xanthophyll cycle carotenoids in the protection of photosynthesis. Trends Plant Sci..

[213] Müller P., Li X.P., Niyogi K.K. (2001). Non-photochemical quenching. A response to excess light energy. Plant Physiol..

[214] Zivcak M., Brestic M., Balatova Z., Drevenakova P., Olsovska K., Kalaji H.M., Yang X., Allakhverdiev S.I. (2013). Photosynthetic electron transport and specific photoprotective responses in wheat leaves under drought stress. Photosynth. Res..

[215] Kalaji H.M., Schansker G., Ladle R.J., Goltsev V., Bosa K., Allakhverdiev S.I., Brestic M., Bussotti F., Calatayud A., Dąbrowski P. (2014). Frequently asked questions about in vivo chlorophyll fluorescence: Practical issues. Photosynth. Res..

[216] Sukhova E., Khlopkov A., Vodeneev V., Sukhov V. (2020). Simulation of a nonphotochemical quenching in plant leaf under different light intensities. Biochim. Biophys. Acta Bioenerg..

[217] Tjus S.E., Møller B.L., Scheller H.V. (1998). Photosystem I is an early target of photoinhibition in barley illuminated at chilling temperatures. Plant Physiol..

[218] Sonoike K. (2011). Photoinhibition of photosystem I. Physiol. Plant..

[219] Tikkanen M., Aro E.M. (2014). Integrative regulatory network of plant thylakoid energy transduction. Trends Plant Sci..

[220] Tikkanen M., Mekala N.R., Aro E.M. (2014). Photosystem II photoinhibition-repair cycle protects Photosystem I from irreversible damage. Biochim. Biophys. Acta.

[221] Allakhverdiev S.I., Nishiyama Y., Takahashi S., Miyairi S., Suzuki I., Murata N. (2005). Systematic analysis of the relation of electron transport and ATP synthesis to the photodamage and repair of photosystem II in Synechocystis. Plant Physiol..

[222] Vanden Berghe T., Linkermann A., Jouan-Lanhouet S., Walczak H., Vandenabeele P. (2014). Regulated necrosis: The expanding network of non-apoptotic cell death pathways. Nat. Rev. Mol. Cell Biol..

[223] Mittler R. (2017). ROS are good. Trends Plant Sci..

[224] Pogány M., von Rad U., Grün S., Dongó A., Pintye A., Simoneau P., Bahnweg G., Kiss L., Barna B., Durner J. (2009). Dual roles of reactive oxygen species and NADPH oxidase RBOHD in an Arabidopsis-Alternaria pathosystem. Plant Physiol..

[225] Sabater B., Martín M. (2013). Hypothesis: Increase of the ratio singlet oxygen plus superoxide radical to hydrogen peroxide changes stress defense response to programmed leaf death. Front. Plant Sci..

[226] Op den Camp R.G., Przybyla D., Ochsenbein C., Laloi C., Kim C., Danon A., Wagner D., Hideg E., Göbel C., Feussner I. (2003). Rapid induction of distinct stress responses after the release of singlet oxygen in Arabidopsis. Plant Cell..

[227] Samuilov V.D., Lagunova E.M., Kiselevsky D.B., Dzyubinskaya E.V., Makarova Y.V., Gusev M.V. (2003). Participation of chloroplasts in plant apoptosis. Biosci. Rep..

[228] Gutiérrez J., González-Pérez S., García-García F., Daly C.T., Lorenzo O., Revuelta J.L., McCabe P.F., Arellano J.B. (2014). Programmed cell death activated by Rose Bengal in Arabidopsis thaliana cell suspension cultures requires functional chloroplasts. J. Exp. Bot..

[229] Mühlenbock P., Szechynska-Hebda M., Plaszczyca M., Baudo M., Mateo A., Mullineaux P.M., Parker J.E., Karpinska B., Karpinski S. (2008). Chloroplast signaling and LESION SIMULATING DISEASE1 regulate crosstalk between light acclimation and immunity in Arabidopsis. Plant Cell..

[230] Mateo A., Mühlenbock P., Rustérucci C., Chang C.C., Miszalski Z., Karpinska B., Parker J.E., Mullineaux P.M., Karpinski S. (2004). LESION SIMULATING DISEASE 1 is required for acclimation to conditions that promote excess excitation energy. Plant Physiol..

[231] Garmier M., Priault P., Vidal G., Driscoll S., Djebbar R., Boccara M., Mathieu C., Foyer C.H., De Paepe R. (2007). Light and oxygen are not required for harpin-induced cell death. J. Biol. Chem..

[232] Colombatti F., Gonzalez D.H., Welchen E. (2014). Plant mitochondria under pathogen attack: A sigh of relief or a last breath?. Mitochondrion.

[233] Dzyubinskaya E.V., Kiselevsky D.B., Lobysheva N.V., Shestak A.A., Samuilov V.D. (2006). Death of stoma guard cells in leaf epidermis under disturbance of energy provision. Biochem. Mosc..

[234] Asai T., Stone J.M., Heard J.E., Kovtun Y., Yorgey P., Sheen J., Ausubel F.M. (2000). Fumonisin B1-induced cell death in arabidopsis protoplasts requires jasmonate-, ethylene-, and salicylate-dependent signaling pathways. Plant Cell..

[235] Toumi I., Moschou P.N., Paschalidis K.A., Bouamama B., Ben Salem-Fnayou A., Ghorbel A.W., Mliki A., Roubelakis-Angelakis K.A. (2010). Abscisic acid signals reorientation of polyamine metabolism to orchestrate stress responses via the polyamine exodus pathway in grapevine. J. Plant Physiol..

[236] Bharath P., Gahir S., Raghavendra A.S. (2021). Abscisic acid-induced stomatal closure: An important component of plant defense against abiotic and biotic stress. Front. Plant Sci..

[237] Li J.H., Fan L.F., Zhao D.J., Zhou Q., Yao J.P., Wang Z.Y., Huang L. (2021). Plant electrical signals: A multidisciplinary challenge. J. Plant Physiol..

